# Arsenic and chromium topsoil levels and cancer mortality in Spain

**DOI:** 10.1007/s11356-016-6806-y

**Published:** 2016-05-30

**Authors:** Olivier Núñez, Pablo Fernández-Navarro, Iván Martín-Méndez, Alejandro Bel-Lan, Juan F. Locutura, Gonzalo López-Abente

**Affiliations:** 1 Environmental and Cancer Epidemiology Unit, National Centre for Epidemiology, Carlos III Institute of Health, Avda. Monforte de Lemos 5, 28029 Madrid, Spain; 2 Consortium for Biomedical Research in Epidemiology and Public Health (CIBER en Epidemiología y Salud Pública (CIBERESP)), Madrid, Spain; 3 Área de Geoquímica y de Recursos Minerales, Instituto Geológico y Minero de España (IGME), Ríos Rosas, 23, 28003 Madrid, Spain

**Keywords:** Cancer mortality, Epidemiology, Spatial data, Geochemistry, INLA, SPDE

## Abstract

Spatio-temporal cancer mortality studies in Spain have revealed patterns for some tumours which display a distribution that is similar across the sexes and persists over time. Such characteristics would be common to tumours that shared risk factors, including the chemical soil composition. The objective of the present study is to assess the association between levels of chromium and arsenic in soil and the cancer mortality. This is an ecological cancer mortality study at municipal level, covering 861,440 cancer deaths in 7917 Spanish mainland towns from 1999 to 2008. Chromium and arsenic topsoil levels (partial extraction) were determined by ICP-MS at 13,317 sampling points. To estimate the effect of these concentrations on mortality, we fitted Besag, York and Mollié models, which included, as explanatory variables, each town’s chromium and arsenic soil levels, estimated by kriging. In addition, we also fitted geostatistical-spatial models including sample locations and town centroids (non-aligned data), using the integrated nested Laplace approximation (INLA) and stochastic partial differential equations (SPDE). All results were adjusted for socio-demographic variables and proximity to industrial emissions. The results showed a statistical association in men and women alike, between arsenic soil levels and mortality due to cancers of the stomach, pancreas, lung and brain and non-Hodgkin’s lymphomas (NHL). Among men, an association was observed with cancers of the prostate, buccal cavity and pharynx, oesophagus, colorectal and kidney. Chromium topsoil levels were associated with mortality among women alone, in cancers of the upper gastrointestinal tract, breast and NHL. Our results suggest that chronic exposure arising from low levels of arsenic and chromium in topsoil could be a potential risk factor for developing cancer.

**Electronic supplementary material:**

The online version of this article (doi:10.1007/s11356-016-6806-y) contains supplementary material, which is available to authorized users.

## Introduction

Spatio-temporal cancer mortality studies and various cancer atlases in Spain (López-Abente et al. 2007, 2014) have revealed geographical patterns for some tumours, which display the following characteristics: (1) there are spatial distribution patterns that are similar in men and women, (2) there are patterns that persist over time, and (3) the determinants of these patterns are very difficult to ascertain. Such characteristics would be common to tumours that shared risk factors which, among other things, included the chemical composition of the soil, since this generally remains stable over time, can contain carcinogens such as heavy metals and affects both sexes indiscriminately. Cancers of the upper gastrointestinal tract (stomach and oesophagus), pancreas, brain, kidney and thyroids all display the above characteristics.

This study was undertaken as a result of this line of reasoning and the fact that the Spanish Geological and Mining Institute (*Instituto Geológico y Minero de España* (*IGME*)) had recently published the “Geochemical Atlas of Spain”, the first geochemical study of surface materials to cover the entire country (Locutura et al. 2012). Our study thus comes within the sphere of geochemical mapping, a discipline that investigates the concentration levels and variability of different chemical elements, as well as their spatial distribution in the territory’s surface materials. In addition, it also seeks to explain the geogenic or anthropogenic factors which influence this distribution. The Geochemical Atlas of Spain not only contains a comprehensive description of the geochemical composition of the soil at two depth levels (horizons of 0–20 and 20–40 cm), but also enabled high-definition maps of the distributions of the various elements and their associations to be plotted, a feature whose utility far outweighs that of mere description (Locutura et al. 2012). Indeed, the map reveals that many of these elements display a singular spatial pattern which, in some cases, visually resembles the distribution of mortality due to certain tumours.

The presence of toxic metals in soil per se, and in soil impacted by mining (Fernández-Navarro et al. 2012), industry (García-Pérez et al. 2007), agriculture and urbanisation, is a major concern for both human health and ecotoxicology (Ranville 2005). High-level exposures to arsenic and heavy metals have been found to be associated with multiple cancer types, including bladder, colon, kidney, liver, lung, skin and prostate, by numerous epidemiological studies (Naujokas et al. 2013). There is far less information, however, on the health effects of low-dose chronic exposure to many trace metals (Centeno et al. 2013); studies on the health effects of metals and metaloids in topsoil belong to this latter category. The few studies that are available were undertaken in cultivated areas treated with xenobiotics, areas with excess incidence of some cancers and areas with known environmental threats (Zhao et al. 2014; Olawoyin et al. 2012; Pearce et al. 2012) or took the form of exploratory ecological studies (Huang et al. 2013; McKinley et al. 2013).

In this context, the aim of this study was to assess the possible association between arsenic and chromium topsoil levels and mortality due to 27 different tumour locations, with the resulting risk estimates being adjusted for socio-demographic variables and proximity to the industrial sources of these pollutants, as possible confounders.

## Material and methods

### Soil sampling and arsenic and chromium analysis

**Fig. 1:**
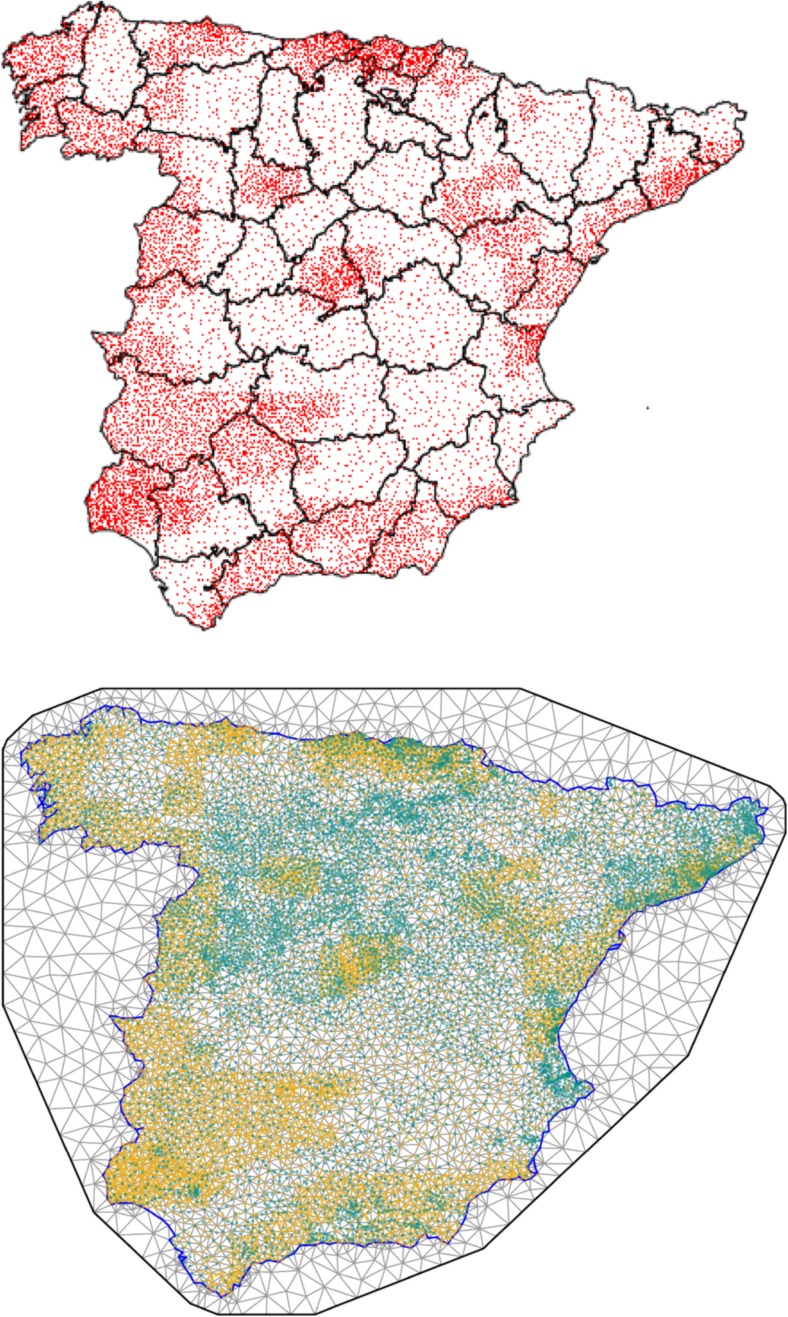
Topsoil sampling sites in mainland Spain (*upper*). Triangulation of mainland Spain (*lower*): *orange points* denote soil sampling locations and *green points*, municipal centroids

Across the period June 2008–November 2010, a total of 21,187 residual soil samples (13,505 from the surface horizon and 7682 from the deeper horizon) were collected at a total of 13,505 sampling points (13,317 in mainland Spain and 188 on the Canary and Balearic islands). Residual soil is a soil belonging to the geological substratum and therefore not transported. Three areas were pinpointed, with different sampling densities defined according to their geological complexity and demographic and industrial density (one sampling point/10 km^2^, one point/20 km^2^ and one point/100 km^2^). Figure 1 (top) shows the different sampling densities adopted and the location of the sampling points.

The residual soil samples (from two horizons, upper and lower) were sieved to 2-mm fraction and then analysed by instrumental inductively coupled plasma mass spectrometry (ICP-MS) after crushing, pulverising and subsequent partial digestion (extraction by aqua regia). For study purposes, we selected the partial extraction results yielded by samples from the upper soil horizon. This choice was due to the fact that, in the event of trace elements being related to possible pollution, this soil sample horizon tends to display the highest trace element content. In the case of chemical analysis with partial extraction, this determination is regarded as being of greater interest for the study of the effect of trace elements on humans, by virtue of its coming closest to the bioavailable content in the sample. Specific bioaccessibility analysis was not undertaken in this study (Barsby et al. 2012; Dean 2010).

A detailed description of the sample collection and the chemical analysis techniques used can be found in the Geochemical Atlas of Spain (Locutura et al. 2012).

### Mortality data

Municipal mortality data (observed cases) were drawn from the records of the National Statistics Institute (NSI) for the study period and corresponded to deaths due to 27 types of malignant tumours (see Supplementary data, Table S1, which shows the list of tumours analysed and their codes as per the International Classification of Diseases-9th and 10th Revisions). Population data were likewise drawn from NSI records. Expected cases were calculated by taking the specific rates for Spain as a whole, broken down by age group (18 groups: 0–4,…, 80–84 years and 85 years and over), sex and 5-year period (1999–2003, 2004–2008) and multiplying these by the person-years for each town, broken down by the same strata. Person-years for each quinquennium were calculated by multiplying the respective populations by 5 (with data corresponding to 2001 and 2006 being taken as the estimator of the population at the midpoint of the study period).

### Statistical analysis

Cancer mortality data are aggregated at a town area level, while the data concentrations of arsenic and chromium in the soil are measures taken at sampling locations across the country (see the Fig. 1, upper). In order to obtain a representative value of this concentration at the area level, an interpolation method (ordinary kriging) was used (Ribeiro and Diggle 2001; Diggle and Ribeiro 2006). The association between metal concentration in the soil and relative risk of cancer mortality was assessed in an ecological regression, where the response was the number of observed deaths from cancer, with expected cases as offset, and the exposure covariate was the kriging estimate of the metal concentration in the municipal-centroid area. This approach (approach A) has the advantage of computational simplicity but ignores the kriging error (Szpiro et al. 2011). Since some areas may contain very few sampling points and metal concentrations may show wide variations, the kriging error can vary substantially from one area to another.

To take this into account, we therefore also adopted a second approach (approach B), whereby spatial variations in metal concentrations (topsoil sampling locations) and in relative risks of cancer mortality (town locations) were jointly modelled and estimated (spatially misaligned data).

Let *expos*_*i*_ denote the logarithm of the metal concentration in soil at each centroid area location *s*_*i*_ and assume for the moment that these concentrations are known. In both approaches, we assume that the observed number of cases *O*_*i*_ in the *i*th area is Poisson distributed, with mean *E*_*i*_*λ*_*i*_, where *E*_*i*_ is the expected number of cases in that area and the relative risk *λ*_*i*_ follows a log-linear model, such that

n.1 $$ \log \left({\lambda}_i\right)=\alpha +\beta expo{s}_i+{u}_i+{v}_i, $$

where *α* is an intercept, *β* is the coefficient for the exposure covariate *expos*_*i*_, *v*_*i*_ is the unstructured normal residual, and *u*_*i*_ is the spatially structured effect which follows an intrinsic conditional autoregressive model, namely, the Besag, York and Mollié model (BYM) (Besag et al. 1991). Inference for the primary parameter of interest *β* is made in a Bayesian framework, and prior distributions are specified for all parameters.

In point of fact, the exposure covariate *expos*_*i*_ is not directly observed. Instead, we observe the metal concentration *c*_*j*_ in soil at sampling locations *s*_*j*_. For these observations, we assume the log-linear model

n.2 $$ \log \left({c}_j\right)= Normal\left({x}_j,{\sigma}_x^2\right), $$

where *x*_*j*_ is the realisation of a Matérn Gaussian field at location *s*_*j*_ and *σ*_*x*_^2^ is a measurement error variance.

In approach A, the value of *x*_*i*_ at each centroid area location *s*_*i*_ is first predicted by ordinary kriging. Then, in a second step, the value of *expos*_*i*_ in the regression (n.1) is replaced by this prediction, and the unknown parameter *β* is estimated. This approach can then be seen as a simple plug-in approach for the unobserved exposure variable *expos*_*i*_. On the other hand, in our second approach (approach B), *expos*_*i*_ is a latent variable equal to *x*_*i*_ and its relationship with the relative risk of mortality is assessed through joint estimation of models (n.1) and (n.2). Hence, the latter approach leads to more conservative confidence intervals, as it takes into account the uncertainty in the exposure variable. Moreover, in approach B, the Gaussian field in model (n.2) was approximated using the stochastic partial differential equation (SPDE) (Lindgren et al. 2011; Lindgren and Rue 2015), as implemented in integrated nested Laplace approximation (R-INLA) (Rue et al. 2009; Rue and Martino 2010). This approach is based on a triangulated mesh of mainland Spain (see Fig. 1, bottom). The choice of the mesh resolution (number of vertices) is a compromise between the accuracy of this approach and computational costs. To solve this trade-off, we used an information criterion based on the greatest length of the triangle edge allowed. For both arsenic and chromium data, the selected value of this length was 5 km. The extension of the mesh with a lower resolution around the Spanish mainland was constructed to control for boundary effects.

In addition to model (n.1), another ecological regression (n.3) was considered to account for potential socio-demographic and environmental confounding factors:

n.3 $$ \log \left({\lambda}_i\right)=\alpha +\beta expo{s}_i+{\displaystyle \sum_j{\delta}_j} So{c}_{ij}+\gamma Indu{s}_i+{u}_i+{v}_i, $$

where the socio-demographic indicators (*Soc*_*ij*_) were obtained from the 1991 census and considered for their availability at the city level and potential explanatory ability vis-à-vis certain geographic mortality patterns (López-Abente et al. 2006). These indicators were as follows: population size (categorised into three levels: 0–2000 [rural zone]; 2000–10,000 [semi-urban zone]; and greater than 10,000 inhabitants [urban zone]); percentages of illiteracy, farmers and unemployment; average number of persons per household; and mean income. The covariate *Indus*_*i*_ indicates the presence (within 5 km) of industries with arsenic or chromium emissions (E-PRTR database, Spanish Ministry of Agriculture and Food and Environment 2007).

In the results shown below, the exposure covariate *expos*_*i*_ was treated as a factor categorised into quartiles where the first quartile was the reference. To find out if there is an increase in relative risk (RR) with exposure levels (trend test), the quartile ordinal was included as continuous variable. However, the above categorisation does not apply to approach B, since this variable must be Gaussian and the log of exposure covariate was used.

Descriptive maps were plotted showing the kriging estimate categorised into quantiles of arsenic and chromium topsoil levels in the respective towns included in the study and, by way of example, maps of the distribution of the standardised mortality ratios for cancer of the oesophagus, smoothed using the BYM model. The distribution of other tumours can be found in López-Abente et al. (2014).

## Results

Across the 10 years of study, a total of 861,440 deaths occurred due to the tumours analysed. Table S1 of the supplementary material shows the distribution of these deaths by sex and cancer site.

The mean topsoil concentrations in towns in the study area are shown in Table 1. Soil levels ranged from 0.50 to 2100 mg kg^−1^ for chromium and from 0.10 to 2510 mg kg^−1^ for arsenic. The interpolation procedure reduced the range of determinations in both elements, basically influencing the extreme values. The proximity of the sources of the pollutants studied had the effect of slightly altering their distribution: the difference in means for these elements between towns with and without emissions in the environs, obtained from their distribution a posteriori (using uninformative priors) (Kruschke 2013), was 1.439 mg kg^−1^ (95 % credibility interval (95 % CI) 1.005–1.862) for arsenic and 1.334 mg kg^−1^ (95 % CI 0.808–1.853) for chromium.

**Table 1:** Study of arsenic and chromium topsoil levels (mg kg^−1^), in interpolation by towns and by strata of proximity to industrial emissions

	*N*	Mean ± SD	Min	P(25)	P(50)	P(75)	Max
All samples (13317)							
As		15.060 ± 40.517	0.100	5.300	9.000	15.300	2510.000
Cr		29.790 ± 45.144	0.500	15.600	23.200	33.800	2100.000
Interpolation by towns							
As	7917	13.790 ± 7.173	1.000	9.106	12.810	16.970	99.370
Cr	7917	26.230 ± 10.161	6.458	20.230	24.990	29.860	243.700
Towns without emissions at <5 km							
As	7037	12.650 ± 7.267	1.000	8.976	12.650	16.710	99.370
Towns with emissions at <5 km							
As	880	14.560 ± 6.330	1.421	9.947	14.560	18.290	42.210
Towns without emissions at <5 km							
Cr	6827	26.043 ± 10.256	6.643	20.140	24.840	29.580	243.700
Towns with emissions at <5 km							
Cr	1090	27.402 ± 9.469	6.458	21.280	25.970	31.830	81.100

*N* number of towns

**Fig. 2:**
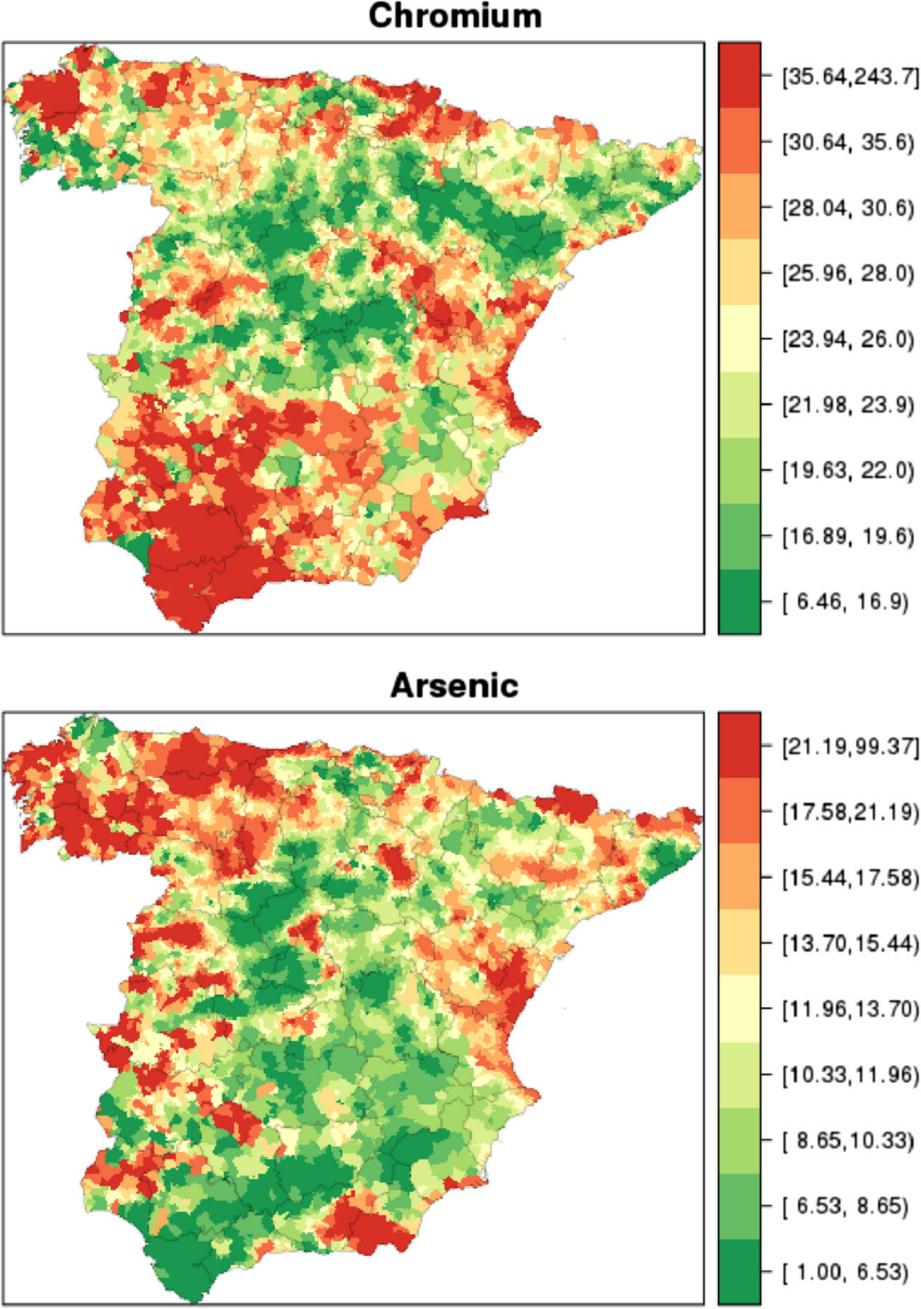
Municipal distribution of chromium and arsenic topsoil concentrations in mainland Spain. Chromium concentrations (mg kg^−1^) (*upper*); arsenic concentrations (mg kg^−1^) (*lower*)

Figure 2 shows the kriging estimate categorised into quantiles of arsenic and chromium topsoil levels in the various towns included in the study. The highest number of towns in the upper quantiles of chromium in soil was mainly observed in the north-west and south-east and at other points in northern and eastern areas of the territory. In the case of arsenic, it was again the northern and eastern areas that registered the highest number of towns in the upper quantiles.

**Fig. 3:**
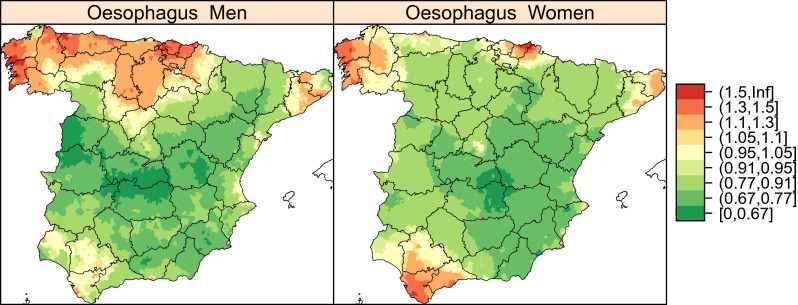
BYM modelling of oesophageal cancer mortality in men (*left*) and women (*right*) over a 10-year period. The maps depict the posterior mean of relative risk for every town. Spain 1999–2008

By way of example, Fig. 3 shows a map depicting the distribution of smoothed relative risk for oesophageal cancer mortality obtained using the BYM model. This map covers the period 1999–2008, and though it shows a pattern displaying similarities between the sexes, attention should nonetheless be drawn to the differences to be seen across a wide area of western Andalusia and in the north of the peninsula.

Tables 2 and 3 show the statistically significant results (marked in italics) of the analyses of association between chromium and arsenic topsoil levels in Spanish towns and mortality due to the selected causes of cancer, for both men and women. Also shown are the RRs and their credibility intervals (95 % CIs) for men and women yielded by the models, i.e., the BYM model with the element as the only explanatory variable (model n.1), and this same model adjusted for socio-demographic variables and proximity to industries that release the pollutant in question into the environment (model n.3). The results of the dose-response analysis (trend test) and those of the models obtained with approach B are given too. The results are reported in their entirety in the supplementary material (Tables S2 and S3).

**Table 2:** Summary of the estimates of the effect (RR) of chromium topsoil levels, categorised in quartiles, on mortality due to different tumour types, by sex

Cancer site	Men						Women					
	Unadjusted			Adjusted			Unadjusted			Adjusted		
	RR	95 %	CI	RR	95 %	CI	RR	95 %	CI	RR	95 %	CI
Buccal cavity and pharynx												
Approach A q2^a^	0.996	0.926–1.071		1.050	0.977–1.128		1.014	0.898–1.144		1.061	0.944–1.190	
q3	0.973	0.901–1.050		1.053	0.981–1.131		0.994	0.879–1.124		1.044	0.931–1.170	
q4	0.947	0.875–1.026		1.025	0.958–1.096		1.105	0.979–1.242		*1.149*	*1.036*–*1.274*	
Trend test^b^	0.981	0.956–1.007		1.006	0.984–1.027		1.033	0.993–1.072		*1.044*	*1.010*–*1.078*	
Approach B SPDE^c^	0.970	0.900–1.037		0.938	0.835–1.024		1.096	0.967–1.233		*1.118*	*1.003*–*1.250*	
Oesophagus												
Approach A q2^a^	0.949	0.883–1.020		0.932	0.867–1.002		0.945	0.798–1.120		0.942	0.796–1.115	
q3	0.883	0.818–0.953		0.889	0.827–0.955		0.993	0.838–1.177		1.021	0.867–1.203	
q4	0.947	0.875–1.025		0.954	0.891–1.021		*1.220*	*1.038*–*1.435*		*1.328*	*1.146*–*1.544*	
Trend test^b^	0.981	0.955–1.006		0.985	0.964–1.007		*1.078*	*1.022*–*1.137*		*1.115*	*1.063*–*1.170*	
Approach B PDE ^c^	0.978	0.893–1.060		0.990	0.901–1.129		1.254	0.989–1.497		1.263	0.991–1.508	
Liver												
Approach A q2^a^	*1.104*	*1.011*–*1.206*		1.003	0.926–1.086		0.973	0.853–1.110		0.864	0.761–0.980	
q3	1.027	0.934–1.130		0.944	0.871–1.022		1.020	0.889–1.172		0.921	0.814–1.041	
q4	1.097	0.992–1.213		0.982	0.911–1.059		1.041	0.902–1.201		0.935	0.833–1.049	
Trend test^b^	1.021	0.989–1.055		0.990	0.967–1.014		1.017	0.971–1.066		0.989	0.953–1.026	
Approach B SPDE^c^	1.026	0.930–1.151		1.051	0.948–1.197		1.050	0.905–1.198		1.020	0.891–1.184	
Larynx												
Approach A q2^a^	0.930	0.864–1.001		0.944	0.879–1.013		1.122	0.871–1.444		1.188	0.909–1.550	
q3	0.965	0.893–1.042		1.009	0.941–1.082		1.116	0.866–1.435		1.206	0.925–1.571	
q4	0.930	0.858–1.008		1.012	0.947–1.081		1.175	0.954–1.453		1.188	0.940–1.507	
Trend test^b^	0.982	0.957–1.008		1.011	0.990–1.032		1.049	0.982–1.121		1.048	0.974–1.127	
Approach B SPDE^c^	0.963	0.827–1.068		0.990	0.905–1.074		*1.279*	*1.040*–*1.567*		*1.295*	*1.037*–*1.611*	
Pleura												
Approach A q2^a^	1.052	0.840–1.318		0.970	0.777–1.209		0.951	0.701–1.291		0.879	0.666–1.157	
q3	1.192	0.944–1.508		1.223	0.990–1.51		1.162	0.866–1.554		*1.312*	*1.020*–*1.686*	
q4	1.099	0.856–1.408		1.046	0.854–1.282		1.273	0.961–1.690		1.239	0.989–1.557	
Trend test^b^	1.037	0.957–1.123		1.030	0.967–1.098		*1.098*	*1.002*–*1.203*		*1.103*	*1.027*–*1.185*	
Approach B SPDE^c^	1.182	0.891–1.515		1.201	0.915–1.746		*1.474*	*1.120*–*1.975*		*1.399*	*1.074*–*1.871*	
Breast												
Approach A q2^a^							1.028	0.986–1.072		*1.042*	*1.003*–*1.081*	
q3							0.992	0.949–1.036		1.015	0.977–1.053	
q4							1.007	0.961–1.054		*1.045*	*1.009*–*1.082*	
Trend test^b^							0.998	0.984–1.013		*1.011*	*1.000*–*1.022*	
Approach B SPDE^c^							0.972	0.927–1.022		0.976	0.934–1.020	
Uterus												
Approach A q2^a^							0.989	0.930–1.051		1.022	0.963–1.086	
q3							0.991	0.930–1.055		1.031	0.971–1.094	
q4							1.046	0.982–1.113		*1.089*	*1.031*–*1.151*	
Trend test^b^							1.016	0.996–1.037		*1.029*	*1.011*–*1.047*	
Approach B SPDE^c^							*1.079*	*1.019*–*1.130*		1.026	0.963–1.092	
Kidney												
Approach A q2^a^	1.061	0.984–1.145		1.063	0.993–1.139		0.952	0.863–1.049		0.931	0.853–1.015	
q3	1.056	0.976–1.142		*1.093*	*1.021*–*1.170*		0.964	0.872–1.065		0.990	0.908–1.077	
q4	1.026	0.945–1.113		1.034	0.969–1.103		1.005	0.911–1.108		0.990	0.917–1.070	
Trend test^b^	1.005	0.979–1.031		1.008	0.988–1.029		1.005	0.974–1.038		1.004	0.980–1.030	
Approach B SPDE^c^	1.009	0.926–1.095		1.005	0.929–1.083		0.981	0.887–1.081		0.980	0.880–1.077	
NHL												
Approach A q2^a^	1.022	0.944–1.106		1.013	0.939–1.092		1.049	0.972–1.132		1.050	0.974–1.131	
q3	1.032	0.952–1.119		1.061	0.986–1.142		1.029	0.953–1.112		1.058	0.982–1.139	
q4	0.994	0.915–1.080		0.987	0.919–1.059		1.076	0.997–1.161		*1.092*	*1.018*–*1.170*	
Trend test^b^	0.997	0.971–1.024		0.997	0.975–1.02		1.021	0.996–1.046		*1.027*	*1.005*–*1.050*	
Approach B SPDE^c^	0.994	0.915–1.080		0.984	0.909–1.067		1.062	0.985–1.132		1.064	0.988–1.143	

The table shows the results of the approaches A and B, broken down as follows: unadjusted (model n.1) and adjusted for socio-demographic variables and industrial emissions (model n.3)

^a^Quartiles: reference [6.46, 20.2], q2 [20.24, 25.0], q3 [24.99, 29.9] and q4 [29.86, 243.7 mg kg^−1^]

^b^RR, taking quartiles as a categorical variable

^c^RR for a change of one unit in the logarithm of the elements’ soil concentration

**Table 3:** Summary of estimates of the effect (RR) of arsenic topsoil levels, categorised in quartiles, on mortality due to different tumour types, by sex

Cancer site	Men						Women					
	Unadjusted			Adjusted			Unadjusted			Adjusted		
	RR	95 %	CI	RR	95 %	CI	RR	95 %	CI	RR	95 %	CI
Buccal cavity and pharynx												
Approach A q2^a^	1.003	0.937–1.073		0.987	0.922–1.057		1.102	0.986–1.233		1.069	0.958–1.194	
q3	1.018	0.943–1.099		1.048	0.977–1.123		1.042	0.918–1.183		1.050	0.931–1.184	
q4	*1.100*	*1.018*–*1.189*		*1.232*	*1.158*–*1.310*		1.076	0.957–1.211		1.051	0.947–1.165	
Trend test^b^	*1.033*	*1.006*–*1.059*		*1.076*	*1.054*–*1.097*		1.018	0.980–1.057		1.012	0.979–1.046	
Approach B SPDE^c^	*1.081*	*1.025*–*1.149*		*1.081*	*1.024*–*1.147*		1.053	0.974–1.144		1.048	0.971–1.137	
Oesophagus												
Approach A q2^a^	0.976	0.910–1.046		0.951	0.887–1.021		0.920	0.789–1.073		0.865	0.738–1.012	
q3	0.959	0.887–1.038		1.004	0.935–1.078		0.912	0.766–1.085		0.925	0.783–1.091	
q4	1.030	0.952–1.115		*1.148*	*1.077*–*1.224*		1.020	0.866–1.198		1.092	0.948–1.26	
Trend test^b^	1.010	0.984–1.037		*1.052*	*1.030*–*1.074*		1.010	0.957–1.066		1.043	0.996–1.093	
Approach B SPDE^c^	1.032	0.978–1.095		1.026	0.971–1.088		1.052	0.938–1.178		1.042	0.926–1.167	
Stomach												
Approach A q2^a^	1.012	0.965–1.062		1.005	0.957–1.056		0.980	0.925–1.039		0.994	0.939–1.053	
q3	1.002	0.947–1.060		1.012	0.962–1.064		0.974	0.911–1.041		0.996	0.939–1.057	
q4	1.054	0.994–1.117		*1.087*	*1.039*–*1.138*		0.990	0.925–1.060		*1.072*	*1.017*–*1.131*	
Trend test^b^	1.017	0.997–1.036		*1.028*	*1.012*–*1.043*		0.997	0.975–1.020		*1.022*	*1.004*–*1.040*	
Approach B SPDE^c^	1.035	0.994–1.079		1.028	0.986–1.073		1.015	0.970–1.069		1.014	0.964–1.064	
Colorectal												
Approach A q2^a^	*1.062*	*1.021*–*1.104*		1.029	0.995–1.065		1.010	0.972–1.05		1.011	0.976–1.047	
q3	*1.051*	*1.006*–*1.098*		*1.050*	*1.014*–*1.087*		0.990	0.948–1.034		1.001	0.966–1.038	
q4	*1.077*	*1.029*–*1.127*		*1.093*	*1.059*–*1.128*		1.004	0.960–1.049		1.004	0.972–1.037	
Trend test^b^	*1.022*	*1.007*–*1.037*		*1.030*	*1.019*–*1.040*		1.000	0.985–1.014		1.000	0.990–1.011	
Approach B SPDE^c^	*1.042*	*1.009*–*1.080*		1.026	0.996–1.057		1.008	0.976–1.039		0.996	0.968–1.026	
Liver												
Approach A q2^a^	0.995	0.913–1.083		0.866	0.801–0.936		1.082	0.957–1.224		0.881	0.781–0.992	
q3	0.987	0.896–1.087		0.907	0.838–0.981		0.953	0.827–1.097		0.863	0.762–0.978	
q4	*1.136*	*1.028*–*1.255*		1.063	0.990–1.141		1.059	0.918–1.223		0.928	0.831–1.036	
Trend test^b^	*1.042*	*1.008*–*1.077*		*1.027*	*1.003*–*1.051*		1.009	0.963–1.058		0.979	0.945–1.015	
Approach B SPDE^c^	*1.087*	*1.013*–*1.180*		1.048	0.979–1.135		0.984	0.893–1.087		0.938	0.856–1.042	
Pancreas												
Approach A q2^a^	1.044	0.991–1.099		1.029	0.979–1.081		1.050	0.992–1.111		1.023	0.971–1.078	
q3	0.989	0.932–1.049		1.001	0.950–1.055		1.029	0.966–1.097		1.040	0.984–1.099	
q4	1.060	0.999–1.124		*1.092*	*1.043*–*1.144*		1.053	0.988–1.122		*1.059*	*1.009*–*1.112*	
Trend test^b^	1.015	0.996–1.035		*1.027*	*1.012*–*1.043*		1.014	0.993–1.035		*1.019*	*1.003*–*1.035*	
Approach B SPDE^c^	*1.042*	*1.000*–*1.095*		1.029	0.991–1.069		1.010	0.966–1.056		1.002	0.961–1.047	
Larynx												
Approach A q2^a^	0.951	0.890–1.016		0.928	0.868–0.991		0.975	0.773–1.227		0.973	0.766–1.233	
q3	0.963	0.891–1.04		0.931	0.868–0.997		0.873	0.665–1.137		0.914	0.689–1.204	
q4	1.013	0.937–1.096		1.014	0.953–1.079		1.096	0.897–1.34		1.040	0.838–1.293	
Trend test^b^	1.006	0.980–1.032		1.007	0.987–1.027		1.028	0.963–1.098		1.013	0.944–1.087	
Approach B SPDE^c^	1.011	0.955–1.068		1.012	0.958–1.071		1.053	0.888–1.216		*1.541*	*1.259*–*1.783*	
Lung												
Approach A q2^a^	0.994	0.962–1.027		0.990	0.961–1.019		*1.072*	*1.003*–*1.146*		1.036	0.977–1.098	
q3	1.001	0.962–1.041		1.004	0.974–1.034		*1.109*	*1.030*–*1.194*		*1.087*	*1.023*–*1.154*	
q4	1.018	0.976–1.062		*1.044*	*1.015*–*1.073*		*1.133*	*1.050*–*1.222*		*1.129*	*1.069*–*1.191*	
Trend test^b^	1.006	0.992–1.021		*1.015*	*1.006*–*1.024*		*1.041*	*1.015*–*1.067*		*1.042*	*1.024*–*1.06*	
Approach B SPDE^c^	1.014	0.982–1.049		1.008	0.978–1.039		*1.058*	*1.001*–*1.114*		1.037	0.976–1.089	
Prostate												
Approach A q2^a^	1.005	0.967–1.044		*1.036*	*1.000*–*1.074*							
q3	0.990	0.948–1.033		1.027	0.989–1.065							
q4	0.984	0.942–1.029		*1.054*	*1.019*–*1.091*							
Trend test^b^	0.994	0.980–1.008		*1.016*	*1.005*–*1.027*							
Approach B SPDE^c^	0.994	0.964–1.025		0.993	0.961–1.023							
Kidney												
Approach A q2^a^	1.051	0.980–1.127		0.983	0.919–1.051		0.969	0.886–1.061		0.933	0.861–1.011	
q3	1.077	0.996–1.166		1.044	0.972–1.119		0.978	0.883–1.082		0.982	0.899–1.073	
q4	*1.132*	*1.047*–*1.223*		*1.094*	*1.027*–*1.164*		0.985	0.894–1.084		0.970	0.900–1.045	
Trend test^b^	*1.041*	*1.015*–*1.068*		*1.035*	*1.014*–*1.056*		0.997	0.966–1.028		0.996	0.973–1.02	
Approach B SPDE^c^	*1.081*	*1.027*–*1.144*		1.052	0.999–1.107		0.995	0.930–1.058		0.982	0.921–1.046	
Brain												
Approach A q2^a^	*1.071*	*1.007*–*1.139*		*1.069*	*1.006*–*1.137*		1.063	0.989–1.142		1.037	0.966–1.114	
q3	1.030	0.962–1.103		1.050	0.983–1.121		1.057	0.975–1.146		1.076	0.998–1.161	
q4	*1.118*	*1.049*–*1.190*		*1.135*	*1.071*–*1.202*		*1.093*	*1.012*–*1.18*		*1.107*	*1.036*–*1.184*	
Trend test^b^	*1.033*	*1.012*–*1.054*		*1.038*	*1.020*–*1.057*		*1.027*	*1.002*–*1.053*		*1.035*	*1.013*–*1.057*	
Approach B SPDE^c^	*1.052*	*1.008*–*1.099*		*1.050*	*1.003*–*1.097*		*1.057*	*1.004*–*1.114*		1.051	0.997–1.107	
NHL												
Approach A q2^a^	1.019	0.945–1.098		0.989	0.920–1.063		*1.086*	*1.012*–*1.165*		1.033	0.962–1.108	
q3	1.003	0.925–1.088		1.017	0.943–1.095		1.069	0.988–1.156		*1.080*	*1.003*–*1.163*	
q4	1.043	0.962–1.129		*1.099*	*1.028*–*1.174*		*1.091*	*1.012*–*1.175*		*1.126*	*1.054*–*1.203*	
Trend test^b^	1.012	0.986–1.039		*1.034*	*1.012*–*1.056*		1.025	1.000–1.049		*1.041*	*1.020*–*1.063*	
Approach B SPDE^c^	1.032	0.976–1.087		1.019	0.961–1.074		1.049	0.998–1.108		1.035	0.986–1.090	
Leukaemias												
Approach A q2^a^	1.030	0.973–1.091		0.995	0.941–1.051		1.024	0.963–1.090		0.985	0.929–1.045	
q3	1.046	0.983–1.114		1.035	0.976–1.097		1.012	0.944–1.085		0.990	0.929–1.056	
q4	*1.061*	*1.000*–*1.126*		1.031	0.978–1.086		1.027	0.964–1.095		0.991	0.935–1.048	
Trend test^b^	*1.019*	*1.000*–*1.039*		1.012	0.996–1.029		1.007	0.987–1.028		0.998	0.980–1.016	
Approach B SPDE^c^	*1.045*	*1.005*–*1.088*		*1.040*	*1.000*–*1.087*		1.011	0.970–1.061		1.003	0.960–1.054	

The table shows the results of the approaches A and B, broken down as follows: unadjusted (model n.1) and adjusted for socio-demographic variables and industrial emissions (model n.3)

^a^Quartiles: reference [1.00, 9.11], q2 [9.11, 12.82], q3 [12.82, 16.97] and q4 [16.97, 99.37 mg kg^−1^]

^b^RR, taking quartiles as a categorical variable

^c^RR for a change of one unit in the logarithm of the elements’ soil concentration

In the case of chromium, no association whatsoever was found in men. In women, however, irrespective of the proximity of chromium-releasing industries, chromium topsoil levels in the upper as opposed to the lower quartile were associated with mortality due to cancer of the buccal cavity and pharynx (RR 1.149, 95 % CI 1.036–1.274), cancer of the oesophagus (1.328, 1.146–1.544), non-Hodgkin’s lymphoma (NHL) (1.092, 1.018–1.170) and breast cancer (1.045, 1.009–1.082). The trend in RR by quartile of chromium concentration was significant for all four tumour sites (trend test).

For arsenic, the towns included in the upper versus the lower quartile of arsenic concentrations (reference) displayed excess mortality due to cancers of the brain, stomach, pancreas and lung and NHL among men and women alike. This association was predominantly observed in the model adjusted for socio-demographic variables and industrial emissions of arsenic, showing a statistically significant increase in RR with the quartiles (trend test). The tumours that showed a statistical association in men only were those of buccal cavity and pharynx, oesophagus and colorectal and kidney cancer. Prostate cancer was also associated with arsenic in soil.

## Discussion

Studies of the geographical distribution of cancer mortality in Spain have revealed the existence of different spatial patterns for different cancer sites that is difficult to explain. The aetiology of malignant tumours is of great complexity, owing to the presence of many determinants of a different nature (environmental, including habits, diet, environs and occupation, biological and genetic), some of which (environmental) are and some of which (biological and genetic) are not linked to the territory.

The results of this study suggest that low bioavailable arsenic levels in soil might give rise to a population exposure that was statistically associated with higher mortality due to cancers of the stomach, pancreas, lung and brain and NHL, among men and women alike. While chromium topsoil levels were associated with higher female mortality due to cancers of the upper gastrointestinal tract (buccal cavity, pharynx and oesophagus), breast cancer and NHL, no such association was found in men.

Arsenic is a known carcinogen in the skin, lung, bladder, liver and kidney, with the evidence suggesting that lung cancer is the most common cause of arsenic-related mortality (IARC 2012). People can be exposed to arsenic in food and water and from inhalation, e.g. breathing sawdust or smoke from burning arsenic-treated wood or fly ash from combustion of As-rich coal (ATSDR 2007).

Current evidence indicating that exposure to arsenic is a risk factor for cancer in the general population comes from occupational studies based on cohorts of workers who inhaled air contaminated by arsenic and other products and from studies in places with populations exposed to high arsenic concentrations in drinking water over prolonged periods of time (Straif et al. 2009). These have highlighted its association with the increase in incidence of lung, bladder, skin, kidney, liver and possibly prostate cancer (Nordstrom 2002). Currently, the greatest interest in the toxicology of arsenic lies in exposure deriving from this substance’s natural presence in food, water and soil. Understanding the environmental levels that could cause public health problems is thus a critical research area (Hughes et al. 2011).

In the USA, a nationwide survey conducted in areas that were judged not to have anthropogenic sources of arsenic reported that natural background concentrations in soil ranged from less than 1 to 97 mg kg^−1^ (Shacklette and Boerngen 1984). According to our study data, the range was very similar, i.e. 1 to 99.4 mg kg^−1^ (Locutura et al. 2012). Owing to low arsenic bioavailability in soil, it is believed that, as compared to intake of naturally occurring arsenic in water and diet, soil arsenic constitutes only a small fraction of intake (Boyce et al. 2008). In the US population, the major food contributors to inorganic As exposure were the following: vegetables (24 %); fruit juices and fruits (18 %); rice (17 %); beer and wine (12 %); and flour, corn and wheat (11 %). Approximately 10 % of total As exposure from foods is in the form of toxic inorganic As (Xue et al. 2010).

Furthermore, the concentration of heavy metals in soil also determines their presence in animal tissue (López Alonso et al. 2002), and the use of biomarkers in cattle has been suggested as a way of monitoring these elements in the environment, since they avoid the problem of bioavailability posed by soil samples.

The small number of studies means that there is very little epidemiological evidence of the association between arsenic topsoil levels and frequency of cancer. However, heavy metal and arsenic topsoil concentrations serve as an indicator of long-term exposure to these elements (Tchounwou et al. 2012). A recent study on arsenic topsoil levels and cancer undertaken in a province in China reported an association with mortality due to cancers of the colon, stomach, kidney, lung and nasopharynx (Chen et al. 2015): this study included 83 towns, 1683 top soil samples and mortality across the period 2005–2010. Although the dimensions of our study were very different, in view of the fact that it covered a 10-year mortality period from 1999 to 2008, included all 7917 towns across mainland Spain and used 13,317 sampling points in estimating arsenic and chromium levels, there is a certain coincidence in terms of the tumour sites for which excess risk was found.

Numerous studies have identified associations between lung cancer and inhaled hexavalent chromium (Cr(VI)) in occupational settings. Furthermore, it is a component of the carcinogenicity of tobacco smoke. Chromium may possibly cause gastrointestinal tract cancer due to drinking Cr-laden water and eating Cr-laden vegetables (Peralta-Videa et al. 2009; Welling et al. 2015). Inhalation of Cr(VI) has occurred in a number of industries, including leather tanning, chrome plating, cement works and stainless steel welding and manufacturing.

It is noteworthy that, in addition to breast cancer, our study observed the association between chromium concentrations and cancers of the buccal cavity, pharynx and oesophagus and NHL exclusively in women. To our knowledge, the origin of this differential risk is unknown, though in the case of cancer of oesophagus, it might be linked to the different geographical mortality pattern. One possible explanation could be that exposure to food and drinking water containing chromium has greater toxicity because it can take place over the long term (e.g., lifetime) and is more likely to occur at particularly susceptible life stages (e.g., in foetuses, children and pregnant women) than in occupational exposures (Welling et al. 2015).

Heavy metal pollution in soil has received much attention because metals are hardly decomposable by soil microbes and can amplify with food chain extension, which in turn poses a potential threat to human health (Li et al. 2014). Human beings could be exposed to heavy metals from vegetable soils via the following six main pathways: (1) direct ingestion of soil particles, (2) dermal contact with soil particles, (3) diet through the food chain, (4) inhalation of soil particles from the air, (5) oral intake from groundwater and (6) dermal intake from groundwater (Abrahams 2002; Liu et al. 2013).

We are unaware of the existence of any study comparable to ours in terms of dimension and scope. Our study encompasses the whole of mainland Spain, contains an estimate of As and Cr topsoil levels for close on 8000 towns obtained from a mesh of more than 13,000 sampling points and covered a broad study period spanning mortality over 10 years. Statistical analysis was performed using hierarchical models with a spatial component (Besag et al. 1991) fitted by R-INLA (Lindgren and Rue 2015). In these models, the risk of falling into the ecological fallacy is minimised by using a very small spatial scale and making no inferences at an individual level (Clayton et al. 1993). Moreover, to account for the spatial interpolation error in the inference, a multivariate model for spatially misaligned data is used (the set of observed locations for the explanatory variable is not identical to that for the response variable) (Cameletti et al. 2013). In this model, the inference is arrived at using the SPDE approach (Lindgren et al. 2011), which makes it computationally feasible and efficient. Although this model only allows to estimate the RR of the variable of exposure as a continuous variable, the estimation in many cases confirms the results of previous analyses and, being more conservative, generally going in the same direction of the association.

Data from soil geochemical studies are usually recorded in parts per million or milligram per kilogram and have been called compositional data requiring specific transformations (Aitchison 1994, 2003). We recognise the compositional/multivariate inherent soil data nature, but this aspect has not been explored in this study. This line has to be developed to a greater extent.

Insofar as limitations are concerned, it should be noted that this was an ecological mortality study with all the problems of using data grouped by town. The study assumed that As and Cr topsoil levels determined each town’s population exposure, and data on possible important confounding variables, such as smoking habit, were lacking. Even so, an effort was made to control for such confounders, by including a series of socio-demographic components as variables of adjustment and by attempting to control for the anthropogenic origin of As and Cr through data on the proximity of the sources of these elements.

Furthermore, it is important to stress that residing in a town with Cr and As levels in the upper quartile in no way implies that their spatial location would in itself give rise to any given cancer. The influence on the population of other socio-demographic and lifestyle factors and other exposures must be borne in mind when it comes to assessing the associations found.

With respect to possible intervention measures, a constant factor when reviewing publications relating to metal and metaloid soil concentration is the warning sounded by researchers as to the importance of controlling and limiting As levels in both soil and, due to its incorporation into the trophic chain, food (Micó et al. 2007; Burló et al. 2012; Muñoz et al. 2000; Peralta-Videa et al. 2009; van Geen et al. 1997; Delgado-Andrade et al. 2003; Peña-Fernández et al. 2014).

To conclude, the results show a statistical association in men and women alike between arsenic topsoil concentration and mortality due to cancers of the stomach, pancreas, lung and brain and NHL. Furthermore, an association was observed with cancers of the buccal cavity and pharynx, colorectal, renal and prostate in men. Chromium topsoil levels were associated with higher mortality in women due to cancer of the upper gastrointestinal tract, breast cancer and NHL, but no such association was found in men.

Access to the data of composition of the soil and its inclusion in epidemiological studies of health in humans is very innovative and opens an important way to try to understand the set of expositions that determine the frequency of cancer and other chronic diseases. On the other hand, the contribution of the geochemical atlas with an entire country geo-coded data is a great contribution to the environmental epidemiology and public health in general.

## Electronic supplementary material

Below is the link to the electronic supplementary material.

ESM 1 (DOC 981 kb)
