# Supplementary material for: Arsenic and chromium topsoil levels and cancer mortality in Spain
Source: Environ Sci Pollut Res Int. 2016 May 30;23(17):17664–75. doi: 10.1007/s11356-016-6806-y (PMC5010621; doi:10.1007/s11356-016-6806-y)
Supplement: Supplementary file 1 — (DOC 981 kb) [file 11356_2016_6806_MOESM1_ESM.doc]

**Supplementary material**

**Arsenic and chromium topsoil levels and cancer mortality in Spain**

Table S1. Number of deaths by different studied tumours produced in continental Spain between the years 1999-2008.

| **Cancer site** | **ICD-9** | **ICD-10** | **Deaths Men** | **Deaths Women** | **Total** |
| --- | --- | --- | --- | --- | --- |
| Buccal cav. and pharynx | C00-C14 | 140-149 | 16274 | 3761 | 20035 |
| Esophagus | C15 | 150 | 14287 | 2228 | 16515 |
| Stomach | C16 | 151 | 34679 | 21692 | 56371 |
| Colorectal | C18-C21 | 153-154,159.0 | 68353 | 51310 | 119663 |
| Liver | C22.0 | 155.0 | 16994 | 6271 | 23265 |
| Gallbladder | C23-C24 | 156 | 4528 | 8005 | 12533 |
| Pancreas | C25 | 157 | 22458 | 19816 | 42274 |
| Peritoneum | C45.1.C48 | 158 | 964 | 1189 | 2153 |
| Nasal cavity | C30-C31 | 160 | 613 | 266 | 879 |
| Larynx | C32 | 161 | 15110 | 609 | 15719 |
| Lung | C33-C34 | 162 | 155142 | 21657 | 176799 |
| Pleura | C38.4,C45.0 | 163 | 1599 | 650 | 2249 |
| Bone | C40-C41 | 170 | 1464 | 1124 | 2588 |
| Connective tissue | C49 | 171 | 2102 | 1957 | 4059 |
| Melanoma | C43 | 172 | 4003 | 3356 | 7359 |
| Skin | C44 | 173 | 2295 | 2035 | 4330 |
| Breast | C50 | 174 | - | 54887 | 54887 |
| Uterus | C53-C55 | 179-182 | - | 17117 | 17117 |
| Ovarian | C56,C57 | 183 | - | 17768 | 17768 |
| Prostate | C61 | 185 | 52528 | - | 52528 |
| Bladder | C67 | 188 | 33632 | 7212 | 40844 |
| Kidney | C64-C66,C68 | 189 | 11509 | 5854 | 17363 |
| Brain | C71 | 191 | 12371 | 9881 | 22252 |
| Thyroid | C73 | 193 | 880 | 1696 | 2576 |
| NHL | C82-C85,C96 | 200,202 | 11706 | 10499 | 22205 |
| Myeloma | C90 | 203 | 7308 | 7450 | 14758 |
| Leukemias | C91-C95 | 204-208 | 15861 | 12376 | 28237 |

Table S2. Summary of the estimates of the effect (RR) of **chromium** **topsoil levels**, categorised in quartiles, on mortality due to different tumour types, by sex. The table shows the results of the approaches A and B, broken down as follows: unadjusted (model n.1); and adjusted for socio-demographic variables and industrial emissions (model n.3).

|  |  | Men | | | | | | | | |  | Women | | | | | | | | |
| --- | --- | --- | --- | --- | --- | --- | --- | --- | --- | --- | --- | --- | --- | --- | --- | --- | --- | --- | --- | --- |
|  |  | Unadjusted | | | |  | Adjusted | | | |  | Unadjusted | | | |  | Adjusted | | | |
| Cancer site |  | RR |  | 95% | CI |  | RR |  | 95% | CI |  | RR |  | 95% | CI |  | RR |  | 95% | CI |
| **Buccal cavity**  **and pharynx** |  |  |  |  |  |  |  |  |  |  |  |  |  |  |  |  |  |  |  |  |
| Approach A q2^a^ |  | 0.996 | xx | 0.926 | 1.071 |  | 1.050 |  | 0.977 | 1.128 |  | 1.014 |  | 0.898 | 1.144 |  | 1.061 |  | 0.944 | 1.19 |
| q3 |  | 0.973 |  | 0.901 | 1.050 |  | 1.053 |  | 0.981 | 1.131 |  | 0.994 |  | 0.879 | 1.124 |  | 1.044 |  | 0.931 | 1.17 |
| q4 |  | 0.947 |  | 0.875 | 1.026 |  | 1.025 |  | 0.958 | 1.096 |  | 1.105 |  | 0.979 | 1.242 |  | **1.149** |  | **1.036** | **1.274** |
| Trend test ^b^ |  | 0.981 |  | 0.956 | 1.007 |  | 1.006 |  | 0.984 | 1.027 |  | 1.033 |  | 0.993 | 1.072 |  | **1.044** |  | **1.010** | **1.078** |
| Approach B SPDE^c^ |  | 0.970 |  | 0.900 | 1.037 |  | 0.938 |  | 0.835 | 1.024 |  | 1.096 |  | 0.967 | 1.233 |  | **1.118** |  | **1.003** | **1.250** |
|  |  |  |  |  |  |  |  |  |  |  |  |  |  |  |  |  |  |  |  |  |
| **Esophagus** |  |  |  |  |  |  |  |  |  |  |  |  |  |  |  |  |  |  |  |  |
| q2 |  | 0.949 |  | 0.883 | 1.02 |  | 0.932 |  | 0.867 | 1.002 |  | 0.945 |  | 0.798 | 1.120 |  | 0.942 |  | 0.796 | 1.115 |
| q3 |  | 0.883 |  | 0.818 | 0.953 |  | 0.889 |  | 0.827 | 0.955 |  | 0.993 |  | 0.838 | 1.177 |  | 1.021 |  | 0.867 | 1.203 |
| q4 |  | 0.947 |  | 0.875 | 1.025 |  | 0.954 |  | 0.891 | 1.021 |  | **1.220** |  | **1.038** | **1.435** |  | **1.328** |  | **1.146** | **1.544** |
| trend |  | 0.981 |  | 0.955 | 1.006 |  | 0.985 |  | 0.964 | 1.007 |  | **1.078** |  | **1.022** | **1.137** |  | **1.115** |  | **1.063** | **1.170** |
| spde |  | 0.978 |  | 0.893 | 1.06 |  | 0.990 |  | 0.901 | 1.129 |  | 1.254 |  | 0.989 | 1.497 |  | 1.263 |  | 0.991 | 1.508 |
|  |  |  |  |  |  |  |  |  |  |  |  |  |  |  |  |  |  |  |  |  |
| **Stomach** |  |  |  |  |  |  |  |  |  |  |  |  |  |  |  |  |  |  |  |  |
| Approach A q2^a^ |  | 0.995 |  | 0.946 | 1.047 |  | 0.971 |  | 0.924 | 1.020 |  | 1.053 |  | 0.992 | 1.118 |  | 1.025 |  | 0.968 | 1.085 |
| q3 |  | 0.995 |  | 0.942 | 1.050 |  | 0.950 |  | 0.904 | 0.998 |  | 1.031 |  | 0.968 | 1.100 |  | 0.959 |  | 0.906 | 1.016 |
| q4 |  | 0.993 |  | 0.937 | 1.052 |  | 0.897 |  | 0.855 | 0.942 |  | 0.979 |  | 0.915 | 1.048 |  | 0.879 |  | 0.832 | 0.929 |
| Trend test ^b^ |  | 0.998 |  | 0.979 | 1.017 |  | 0.965 |  | 0.951 | 0.980 |  | 0.990 |  | 0.968 | 1.012 |  | 0.954 |  | 0.938 | 0.971 |
| Approach B SPDE^c^ |  | 0.982 |  | 0.920 | 1.047 |  | 0.986 |  | 0.926 | 1.050 |  | 0.972 |  | 0.906 | 1.057 |  | 0.977 |  | 0.909 | 1.063 |
|  |  |  |  |  |  |  |  |  |  |  |  |  |  |  |  |  |  |  |  |  |
| **Colorectal** |  |  |  |  |  |  |  |  |  |  |  |  |  |  |  |  |  |  |  |  |
| Approach A q2^a^ |  | 0.996 |  | 0.956 | 1.037 |  | 1.019 |  | 0.983 | 1.055 |  | 1.018 |  | 0.977 | 1.061 |  | 1.029 |  | 0.992 | 1.067 |
| q3 |  | 0.991 |  | 0.95 | 1.035 |  | 1.024 |  | 0.989 | 1.06 |  | 0.985 |  | 0.943 | 1.028 |  | 0.996 |  | 0.961 | 1.033 |
| q4 |  | 0.991 |  | 0.946 | 1.038 |  | 1.012 |  | 0.978 | 1.046 |  | 0.999 |  | 0.955 | 1.044 |  | 0.999 |  | 0.965 | 1.034 |
| Trend test ^b^ |  | 0.997 |  | 0.982 | 1.012 |  | 1.003 |  | 0.993 | 1.014 |  | 0.996 |  | 0.982 | 1.011 |  | 0.996 |  | 0.985 | 1.007 |
| Approach B SPDE^c^ |  | 1.007 |  | 0.956 | 1.056 |  | 1.006 |  | 0.963 | 1.051 |  | 0.988 |  | 0.941 | 1.034 |  | 0.988 |  | 0.948 | 1.032 |
|  |  |  |  |  |  |  |  |  |  |  |  |  |  |  |  |  |  |  |  |  |
| **Liver** |  |  |  |  |  |  |  |  |  |  |  |  |  |  |  |  |  |  |  |  |
| Approach A q2^a^ |  | **1.104** |  | **1.011** | **1.206** |  | 1.003 |  | 0.926 | 1.086 |  | 0.973 |  | 0.853 | 1.11 |  | 0.864 |  | 0.761 | 0.98 |
| q3 |  | 1.027 |  | 0.934 | 1.130 |  | 0.944 |  | 0.871 | 1.022 |  | 1.020 |  | 0.889 | 1.172 |  | 0.921 |  | 0.814 | 1.041 |
| q4 |  | 1.097 |  | 0.992 | 1.213 |  | 0.982 |  | 0.911 | 1.059 |  | 1.041 |  | 0.902 | 1.201 |  | 0.935 |  | 0.833 | 1.049 |
| Trend test ^b^ |  | 1.021 |  | 0.989 | 1.055 |  | 0.99 |  | 0.967 | 1.014 |  | 1.017 |  | 0.971 | 1.066 |  | 0.989 |  | 0.953 | 1.026 |
| Approach B SPDE^c^ |  | 1.026 |  | 0.930 | 1.151 |  | 1.051 |  | 0.948 | 1.197 |  | 1.050 |  | 0.905 | 1.198 |  | 1.020 |  | 0.891 | 1.184 |
|  |  |  |  |  |  |  |  |  |  |  |  |  |  |  |  |  |  |  |  |  |
| **Gallbladder** |  |  |  |  |  |  |  |  |  |  |  |  |  |  |  |  |  |  |  |  |
| Approach A q2^a^ |  | 1.065 |  | 0.953 | 1.190 |  | 0.975 |  | 0.871 | 1.110 |  | 1.029 |  | 0.938 | 1.129 |  | 1.006 |  | 0.974 | 1.040 |
| q3 |  | 1.086 |  | 0.970 | 1.216 |  | 0.979 |  | 0.888 | 1.102 |  | 0.994 |  | 0.902 | 1.096 |  | 1.025 |  | 0.942 | 1.116 |
| q4 |  | 1.044 |  | 0.931 | 1.169 |  | 1.020 |  | 0.913 | 1.138 |  | 0.968 |  | 0.876 | 1.069 |  | 1.000 |  | 0.919 | 1.089 |
| Trend test ^b^ |  | 1.012 |  | 0.975 | 1.049 |  | 1.015 |  | 0.914 | 1.126 |  | 0.985 |  | 0.954 | 1.018 |  | 0.989 |  | 0.964 | 1.014 |
| Approach B SPDE^c^ |  | 0.984 |  | 0.876 | 1.107 |  | 1.076 |  | 0.964 | 1.200 |  | 0.981 |  | 0.880 | 1.084 |  | 0.977 |  | 0.901 | 1.059 |
|  |  |  |  |  |  |  |  |  |  |  |  |  |  |  |  |  |  |  |  |  |
| **Pancreas** |  |  |  |  |  |  |  |  |  |  |  |  |  |  |  |  |  |  |  |  |
| Approach A q2^a^ |  | 1.011 |  | 0.956 | 1.069 |  | 1.007 |  | 0.955 | 1.061 |  | 1.009 |  | 0.95 | 1.072 |  | 1.024 |  | 0.969 | 1.082 |
| q3 |  | 1.009 |  | 0.952 | 1.070 |  | 0.996 |  | 0.945 | 1.049 |  | 0.975 |  | 0.915 | 1.038 |  | 0.992 |  | 0.939 | 1.047 |
| q4 |  | 1.042 |  | 0.981 | 1.107 |  | 1.006 |  | 0.958 | 1.057 |  | 0.978 |  | 0.917 | 1.043 |  | 0.987 |  | 0.937 | 1.039 |
| Trend test ^b^ |  | 1.013 |  | 0.994 | 1.033 |  | 1.001 |  | 0.986 | 1.017 |  | 0.990 |  | 0.970 | 1.011 |  | 0.992 |  | 0.976 | 1.009 |
| Approach B SPDE^c^ |  | 1.035 |  | 0.974 | 1.106 |  | 1.028 |  | 0.972 | 1.091 |  | 0.967 |  | 0.904 | 1.035 |  | 0.972 |  | 0.911 | 1.037 |
|  |  |  |  |  |  |  |  |  |  |  |  |  |  |  |  |  |  |  |  |  |
|  |  |  |  |  |  |  |  |  |  |  |  |  |  |  |  |  |  |  |  |  |
| **Peritoneum** |  |  |  |  |  |  |  |  |  |  |  |  |  |  |  |  |  |  |  |  |
| Approach A q2^a^ |  | 1.01 |  | 0.802 | 1.271 |  | 0.991 |  | 0.784 | 1.254 |  | 0.755 |  | 0.617 | 0.92 |  | 0.811 |  | 0.671 | 0.979 |
| q3 |  | 1.125 |  | 0.901 | 1.406 |  | 1.134 |  | 0.904 | 1.425 |  | 0.750 |  | 0.612 | 0.915 |  | 0.821 |  | 0.681 | 0.989 |
| q4 |  | 0.966 |  | 0.788 | 1.186 |  | 0.930 |  | 0.748 | 1.157 |  | 0.834 |  | 0.696 | 1.000 |  | 0.889 |  | 0.756 | 1.046 |
| Trend test ^b^ |  | 0.993 |  | 0.932 | 1.058 |  | 0.984 |  | 0.92 | 1.053 |  | 0.953 |  | 0.898 | 1.012 |  | 0.972 |  | 0.922 | 1.024 |
| Approach B SPDE^c^ |  | 0.945 |  | 0.730 | 1.146 |  | 0.911 |  | 0.722 | 1.118 |  | 0.940 |  | 0.787 | 1.135 |  | 0.927 |  | 0.762 | 1.115 |
|  |  |  |  |  |  |  |  |  |  |  |  |  |  |  |  |  |  |  |  |  |
| **Nasal cavity** |  |  |  |  |  |  |  |  |  |  |  |  |  |  |  |  |  |  |  |  |
| Approach A q2^a^ |  | 0.786 |  | 0.591 | 1.041 |  | 0.813 |  | 0.606 | 1.084 |  | 1.079 |  | 0.755 | 1.534 |  | 1.140 |  | 0.78 | 1.66 |
| q3 |  | 0.972 |  | 0.739 | 1.275 |  | 1.037 |  | 0.79 | 1.358 |  | 1.06 |  | 0.742 | 1.507 |  | 1.155 |  | 0.791 | 1.679 |
| q4 |  | 0.955 |  | 0.739 | 1.232 |  | 0.981 |  | 0.758 | 1.267 |  | 0.799 |  | 0.584 | 1.096 |  | 0.795 |  | 0.557 | 1.137 |
| Trend test ^b^ |  | 1.007 |  | 0.926 | 1.093 |  | 1.02 |  | 0.94 | 1.105 |  | 0.928 |  | 0.841 | 1.023 |  | 0.923 |  | 0.827 | 1.029 |
| Approach B SPDE^c^ |  | 1.124 |  | 0.873 | 1.403 |  | 1.157 |  | 0.877 | 1.468 |  | 0.900 |  | 0.670 | 1.202 |  | 0.928 |  | 0.660 | 1.329 |
|  |  |  |  |  |  |  |  |  |  |  |  |  |  |  |  |  |  |  |  |  |
| **Larynx** |  |  |  |  |  |  |  |  |  |  |  |  |  |  |  |  |  |  |  |  |
| Approach A q2^a^ |  | 0.930 |  | 0.864 | 1.001 |  | 0.944 |  | 0.879 | 1.013 |  | 1.122 |  | 0.871 | 1.444 |  | 1.188 |  | 0.909 | 1.55 |
| q3 |  | 0.965 |  | 0.893 | 1.042 |  | 1.009 |  | 0.941 | 1.082 |  | 1.116 |  | 0.866 | 1.435 |  | 1.206 |  | 0.925 | 1.571 |
| q4 |  | 0.930 |  | 0.858 | 1.008 |  | 1.012 |  | 0.947 | 1.081 |  | 1.175 |  | 0.954 | 1.453 |  | 1.188 |  | 0.94 | 1.507 |
| Trend test ^b^ |  | 0.982 |  | 0.957 | 1.008 |  | 1.011 |  | 0.99 | 1.032 |  | 1.049 |  | 0.982 | 1.121 |  | 1.048 |  | 0.974 | 1.127 |
| Approach B SPDE^c^ |  | 0.963 |  | 0.827 | 1.068 |  | 0.99 |  | 0.905 | 1.074 |  | **1.279** |  | **1.04** | **1.567** |  | **1.295** |  | **1.037** | **1.611** |
|  |  |  |  |  |  |  |  |  |  |  |  |  |  |  |  |  |  |  |  |  |
| **Lung** |  |  |  |  |  |  |  |  |  |  |  |  |  |  |  |  |  |  |  |  |
| Approach A q2^a^ |  | 0.999 |  | 0.965 | 1.034 |  | 0.993 |  | 0.963 | 1.024 |  | 1.012 |  | 0.943 | 1.085 |  | 1.015 |  | 0.955 | 1.079 |
| q3 |  | 0.999 |  | 0.962 | 1.038 |  | 1.015 |  | 0.985 | 1.047 |  | 1.009 |  | 0.938 | 1.086 |  | 1.029 |  | 0.969 | 1.092 |
| q4 |  | 0.951 |  | 0.912 | 0.992 |  | 1.013 |  | 0.983 | 1.044 |  | 1.019 |  | 0.943 | 1.101 |  | 1.049 |  | 0.991 | 1.11 |
| Trend test ^b^ |  | 0.984 |  | 0.97 | 0.997 |  | 1.006 |  | 0.996 | 1.016 |  | 1.006 |  | 0.981 | 1.031 |  | 1.016 |  | 0.998 | 1.034 |
| Approach B SPDE^c^ |  | 0.954 |  | 0.909 | 1.003 |  | 0.972 |  | 0.929 | 1.015 |  | 1.026 |  | 0.938 | 1.11 |  | 1.044 |  | 0.96 | 1.121 |
|  |  |  |  |  |  |  |  |  |  |  |  |  |  |  |  |  |  |  |  |  |
| **Pleura** |  |  |  |  |  |  |  |  |  |  |  |  |  |  |  |  |  |  |  |  |
| Approach A q2^a^ |  | 1.052 |  | 0.84 | 1.318 |  | 0.970 |  | 0.777 | 1.209 |  | 0.951 |  | 0.701 | 1.291 |  | 0.879 |  | 0.666 | 1.157 |
| q3 |  | 1.192 |  | 0.944 | 1.508 |  | 1.223 |  | 0.99 | 1.51 |  | 1.162 |  | 0.866 | 1.554 |  | **1.312** |  | **1.020** | **1.686** |
| q4 |  | 1.099 |  | 0.856 | 1.408 |  | 1.046 |  | 0.854 | 1.282 |  | 1.273 |  | 0.961 | 1.69 |  | 1.239 |  | 0.989 | 1.557 |
| Trend test ^b^ |  | 1.037 |  | 0.957 | 1.123 |  | 1.030 |  | 0.967 | 1.098 |  | **1.098** |  | **1.002** | **1.203** |  | **1.103** |  | **1.027** | **1.185** |
| Approach B SPDE^c^ |  | 1.182 |  | 0.891 | 1.515 |  | 1.201 |  | 0.915 | 1.746 |  | **1.474** |  | **1.120** | **1.975** |  | **1.399** |  | **1.074** | **1.871** |
|  |  |  |  |  |  |  |  |  |  |  |  |  |  |  |  |  |  |  |  |  |
| **Bone** |  |  |  |  |  |  |  |  |  |  |  |  |  |  |  |  |  |  |  |  |
| Approach A q2^a^ |  | 0.982 |  | 0.837 | 1.151 |  | 1.002 |  | 0.849 | 1.183 |  | 1.049 |  | 0.872 | 1.259 |  | 1.084 |  | 0.894 | 1.312 |
| q3 |  | 0.941 |  | 0.801 | 1.103 |  | 0.961 |  | 0.813 | 1.134 |  | 1.058 |  | 0.88 | 1.269 |  | 1.087 |  | 0.898 | 1.315 |
| q4 |  | 0.974 |  | 0.853 | 1.114 |  | 0.986 |  | 0.85 | 1.144 |  | 1.052 |  | 0.902 | 1.227 |  | 1.099 |  | 0.926 | 1.304 |
| Trend test ^b^ |  | 0.991 |  | 0.949 | 1.034 |  | 0.993 |  | 0.947 | 1.041 |  | 1.015 |  | 0.966 | 1.066 |  | 1.027 |  | 0.973 | 1.084 |
| Approach B SPDE^c^ |  | 1.003 |  | 0.882 | 1.140 |  | 1.019 |  | 0.882 | 1.179 |  | 1.051 |  | 0.910 | 1.221 |  | 1.117 |  | 0.947 | 1.319 |
|  |  |  |  |  |  |  |  |  |  |  |  |  |  |  |  |  |  |  |  |  |
| **Connective tissue** |  |  |  |  |  |  |  |  |  |  |  |  |  |  |  |  |  |  |  |  |
| Approach A q2^a^ |  | 1.047 |  | 0.908 | 1.209 |  | 1.033 |  | 0.9 | 1.185 |  | 1.031 |  | 0.877 | 1.209 |  | 1.031 |  | 0.87 | 1.217 |
| q3 |  | 0.961 |  | 0.83 | 1.112 |  | 0.975 |  | 0.848 | 1.12 |  | 0.958 |  | 0.811 | 1.129 |  | 0.967 |  | 0.816 | 1.142 |
| q4 |  | 0.999 |  | 0.876 | 1.143 |  | 0.988 |  | 0.873 | 1.119 |  | 0.979 |  | 0.837 | 1.145 |  | 0.999 |  | 0.854 | 1.167 |
| Trend test ^b^ |  | 0.993 |  | 0.952 | 1.036 |  | 0.991 |  | 0.953 | 1.031 |  | 0.988 |  | 0.939 | 1.038 |  | 0.994 |  | 0.947 | 1.043 |
| Approach B SPDE^c^ |  | 1.004 |  | 0.887 | 1.133 |  | 0.989 |  | 0.877 | 1.121 |  | 1.012 |  | 0.875 | 1.171 |  | 1.028 |  | 0.882 | 1.210 |
|  |  |  |  |  |  |  |  |  |  |  |  |  |  |  |  |  |  |  |  |  |
| **Melanoma** |  |  |  |  |  |  |  |  |  |  |  |  |  |  |  |  |  |  |  |  |
| Approach A q2^a^ |  | 0.998 |  | 0.891 | 1.12 |  | 1.017 |  | 0.92 | 1.125 |  | 1.015 |  | 0.903 | 1.142 |  | 1.058 |  | 0.947 | 1.182 |
| q3 |  | 0.93 |  | 0.826 | 1.049 |  | 0.954 |  | 0.861 | 1.057 |  | 1.028 |  | 0.914 | 1.159 |  | 1.089 |  | 0.976 | 1.215 |
| q4 |  | 0.989 |  | 0.882 | 1.109 |  | 0.999 |  | 0.912 | 1.095 |  | 0.98 |  | 0.876 | 1.103 |  | 0.999 |  | 0.904 | 1.106 |
| Trend test ^b^ |  | 0.993 |  | 0.957 | 1.03 |  | 0.996 |  | 0.967 | 1.025 |  | 0.993 |  | 0.958 | 1.031 |  | 0.998 |  | 0.967 | 1.03 |
| Approach B SPDE^c^ |  | 0.995 |  | 0.884 | 1.108 |  | 0.998 |  | 0.894 | 1.163 |  | 0.950 |  | 0.873 | 1.050 |  | 0.990 |  | 0.811 | 1.129 |
|  |  |  |  |  |  |  |  |  |  |  |  |  |  |  |  |  |  |  |  |  |
| **Skin** |  |  |  |  |  |  |  |  |  |  |  |  |  |  |  |  |  |  |  |  |
| Approach A q2^a^ |  | 0.995 |  | 0.853 | 1.161 |  | 0.978 |  | 0.841 | 1.136 |  | 1.006 |  | 0.847 | 1.193 |  | 1.045 |  | 0.893 | 1.222 |
| q3 |  | 1.046 |  | 0.894 | 1.224 |  | 1.042 |  | 0.899 | 1.207 |  | 1.028 |  | 0.861 | 1.226 |  | 1.056 |  | 0.904 | 1.232 |
| q4 |  | 1.012 |  | 0.865 | 1.184 |  | 0.999 |  | 0.868 | 1.15 |  | 0.965 |  | 0.806 | 1.155 |  | 0.950 |  | 0.818 | 1.102 |
| Trend test ^b^ |  | 1.007 |  | 0.958 | 1.059 |  | 1.004 |  | 0.961 | 1.05 |  | 0.989 |  | 0.933 | 1.047 |  | 0.981 |  | 0.936 | 1.027 |
| Approach B SPDE^c^ |  | 0.968 |  | 0.833 | 1.116 |  | 0.998 |  | 0.861 | 1.187 |  | 0.885 |  | 0.750 | 1.075 |  | 0.847 |  | 0.742 | 0.992 |
|  |  |  |  |  |  |  |  |  |  |  |  |  |  |  |  |  |  |  |  |  |
| **Breast** |  |  |  |  |  |  |  |  |  |  |  |  |  |  |  |  |  |  |  |  |
| Approach A q2^a^ |  |  |  |  |  |  |  |  |  |  |  | 1.028 |  | 0.986 | 1.072 |  | **1.042** |  | **1.003** | **1.081** |
| q3 |  |  |  |  |  |  |  |  |  |  |  | 0.992 |  | 0.949 | 1.036 |  | 1.015 |  | 0.977 | 1.053 |
| q4 |  |  |  |  |  |  |  |  |  |  |  | 1.007 |  | 0.961 | 1.054 |  | **1.045** |  | **1.009** | **1.082** |
| Trend test ^b^ |  |  |  |  |  |  |  |  |  |  |  | 0.998 |  | 0.984 | 1.013 |  | **1.011** |  | **1.000** | **1.022** |
| Approach B SPDE^c^ |  |  |  |  |  |  |  |  |  |  |  | 0.972 |  | 0.927 | 1.022 |  | 0.976 |  | 0.934 | 1.02 |
|  |  |  |  |  |  |  |  |  |  |  |  |  |  |  |  |  |  |  |  |  |
| **Uterus** |  |  |  |  |  |  |  |  |  |  |  |  |  |  |  |  |  |  |  |  |
| Approach A q2^a^ |  |  |  |  |  |  |  |  |  |  |  | 0.989 |  | 0.93 | 1.051 |  | 1.022 |  | 0.963 | 1.086 |
| q3 |  |  |  |  |  |  |  |  |  |  |  | 0.991 |  | 0.93 | 1.055 |  | 1.031 |  | 0.971 | 1.094 |
| q4 |  |  |  |  |  |  |  |  |  |  |  | 1.046 |  | 0.982 | 1.113 |  | **1.089** |  | **1.031** | **1.151** |
| Trend test ^b^ |  |  |  |  |  |  |  |  |  |  |  | 1.016 |  | 0.996 | 1.037 |  | **1.029** |  | **1.011** | **1.047** |
| Approach B SPDE^c^ |  |  |  |  |  |  |  |  |  |  |  | **1.079** |  | **1.019** | **1.130** |  | 1.026 |  | 0.963 | 1.092 |
|  |  |  |  |  |  |  |  |  |  |  |  |  |  |  |  |  |  |  |  |  |
| **Ovarian** |  |  |  |  |  |  |  |  |  |  |  |  |  |  |  |  |  |  |  |  |
| Approach A q2^a^ |  |  |  |  |  |  |  |  |  |  |  | 1.01 |  | 0.951 | 1.071 |  | 1.015 |  | 0.959 | 1.074 |
| q3 |  |  |  |  |  |  |  |  |  |  |  | 0.99 |  | 0.931 | 1.051 |  | 1.003 |  | 0.948 | 1.062 |
| q4 |  |  |  |  |  |  |  |  |  |  |  | 1.016 |  | 0.958 | 1.077 |  | 1.020 |  | 0.967 | 1.075 |
| Trend test ^b^ |  |  |  |  |  |  |  |  |  |  |  | 1.004 |  | 0.985 | 1.022 |  | 1.005 |  | 0.989 | 1.022 |
| Approach B SPDE^c^ |  |  |  |  |  |  |  |  |  |  |  | 1.001 |  | 0.949 | 1.066 |  | 1.008 |  | 0.948 | 1.072 |
|  |  |  |  |  |  |  |  |  |  |  |  |  |  |  |  |  |  |  |  |  |
| **Prostate** |  |  |  |  |  |  |  |  |  |  |  |  |  |  |  |  |  |  |  |  |
| Approach A q2^a^ |  | 0.95 |  | 0.913 | 0.989 |  | 0.962 |  | 0.927 | 0.999 |  |  |  |  |  |  |  |  |  |  |
| q3 |  | 0.965 |  | 0.926 | 1.006 |  | 0.978 |  | 0.943 | 1.015 |  |  |  |  |  |  |  |  |  |  |
| q4 |  | 0.97 |  | 0.928 | 1.013 |  | 0.992 |  | 0.958 | 1.028 |  |  |  |  |  |  |  |  |  |  |
| Trend test ^b^ |  | 0.993 |  | 0.979 | 1.007 |  | 1.000 |  | 0.989 | 1.011 |  |  |  |  |  |  |  |  |  |  |
| Approach B SPDE^c^ |  | 0.995 |  | 0.949 | 1.041 |  | 1.004 |  | 0.957 | 1.049 |  |  |  |  |  |  |  |  |  |  |
|  |  |  |  |  |  |  |  |  |  |  |  |  |  |  |  |  |  |  |  |  |
| **Bladder** |  |  |  |  |  |  |  |  |  |  |  |  |  |  |  |  |  |  |  |  |
| Approach A q2^a^ |  | 1.018 |  | 0.965 | 1.074 |  | 1.018 |  | 0.970 | 1.068 |  | 1.051 |  | 0.960 | 1.150 |  | 1.059 |  | 0.978 | 1.145 |
| q3 |  | 1.005 |  | 0.949 | 1.064 |  | 1.012 |  | 0.965 | 1.061 |  | 1.020 |  | 0.929 | 1.121 |  | 1.037 |  | 0.958 | 1.121 |
| q4 |  | 0.969 |  | 0.912 | 1.029 |  | 1.031 |  | 0.985 | 1.079 |  | 0.995 |  | 0.906 | 1.094 |  | 1.019 |  | 0.948 | 1.095 |
| Trend test ^b^ |  | 0.988 |  | 0.969 | 1.008 |  | 1.009 |  | 0.994 | 1.024 |  | 0.994 |  | 0.965 | 1.024 |  | 1.002 |  | 0.979 | 1.024 |
| Approach B SPDE^c^ |  | 0.962 |  | 0.893 | 1.025 |  | 0.964 |  | 0.91 | 1.023 |  | 1.012 |  | 0.924 | 1.125 |  | 1.009 |  | 0.93 | 1.098 |
|  |  |  |  |  |  |  |  |  |  |  |  |  |  |  |  |  |  |  |  |  |
| **Kidney** |  |  |  |  |  |  |  |  |  |  |  |  |  |  |  |  |  |  |  |  |
| Approach A q2^a^ |  | 1.061 |  | 0.984 | 1.145 |  | 1.063 |  | 0.993 | 1.139 |  | 0.952 |  | 0.863 | 1.049 |  | 0.931 |  | 0.853 | 1.015 |
| q3 |  | 1.056 |  | 0.976 | 1.142 |  | **1.093** |  | **1.021** | **1.170** |  | 0.964 |  | 0.872 | 1.065 |  | 0.990 |  | 0.908 | 1.077 |
| q4 |  | 1.026 |  | 0.945 | 1.113 |  | 1.034 |  | 0.969 | 1.103 |  | 1.005 |  | 0.911 | 1.108 |  | 0.990 |  | 0.917 | 1.07 |
| Trend test ^b^ |  | 1.005 |  | 0.979 | 1.031 |  | 1.008 |  | 0.988 | 1.029 |  | 1.005 |  | 0.974 | 1.038 |  | 1.004 |  | 0.980 | 1.03 |
| Approach B SPDE^c^ |  | 1.009 |  | 0.926 | 1.095 |  | 1.005 |  | 0.929 | 1.083 |  | 0.981 |  | 0.887 | 1.081 |  | 0.980 |  | 0.880 | 1.077 |
|  |  |  |  |  |  |  |  |  |  |  |  |  |  |  |  |  |  |  |  |  |
| **Brain** |  |  |  |  |  |  |  |  |  |  |  |  |  |  |  |  |  |  |  |  |
| Approach A q2^a^ |  | 1.004 |  | 0.939 | 1.073 |  | 1.010 |  | 0.947 | 1.076 |  | 1.005 |  | 0.929 | 1.087 |  | 1.005 |  | 0.932 | 1.083 |
| q3 |  | 0.956 |  | 0.893 | 1.023 |  | 0.964 |  | 0.904 | 1.027 |  | 1.038 |  | 0.958 | 1.124 |  | 1.073 |  | 0.997 | 1.155 |
| q4 |  | 1.022 |  | 0.958 | 1.091 |  | 1.002 |  | 0.945 | 1.063 |  | 1.011 |  | 0.932 | 1.095 |  | 1.022 |  | 0.953 | 1.096 |
| Trend test ^b^ |  | 1.005 |  | 0.984 | 1.026 |  | 0.998 |  | 0.98 | 1.017 |  | 1.005 |  | 0.979 | 1.031 |  | 1.011 |  | 0.989 | 1.033 |
| Approach B SPDE^c^ |  | 1.014 |  | 0.952 | 1.089 |  | 1.007 |  | 0.943 | 1.072 |  | 1.059 |  | 0.977 | 1.146 |  | 1.066 |  | 0.988 | 1.146 |
|  |  |  |  |  |  |  |  |  |  |  |  |  |  |  |  |  |  |  |  |  |
| **Thyroid** |  |  |  |  |  |  |  |  |  |  |  |  |  |  |  |  |  |  |  |  |
| Approach A q2^a^ |  | 0.958 |  | 0.777 | 1.177 |  | 0.978 |  | 0.793 | 1.203 |  | 0.843 |  | 0.711 | 0.997 |  | 0.846 |  | 0.710 | 1.004 |
| q3 |  | 0.935 |  | 0.758 | 1.151 |  | 0.957 |  | 0.777 | 1.177 |  | 0.926 |  | 0.781 | 1.094 |  | 0.953 |  | 0.804 | 1.124 |
| q4 |  | 0.851 |  | 0.706 | 1.025 |  | 0.868 |  | 0.718 | 1.050 |  | 0.961 |  | 0.821 | 1.121 |  | 0.974 |  | 0.836 | 1.133 |
| Trend test ^b^ |  | 0.949 |  | 0.896 | 1.006 |  | 0.954 |  | 0.899 | 1.013 |  | 1.001 |  | 0.951 | 1.052 |  | 1.010 |  | 0.966 | 1.056 |
| Approach B SPDE^c^ |  | 0.893 |  | 0.753 | 1.058 |  | 0.905 |  | 0.754 | 1.100 |  | 1.021 |  | 0.877 | 1.194 |  | 1.032 |  | 0.880 | 1.204 |
|  |  |  |  |  |  |  |  |  |  |  |  |  |  |  |  |  |  |  |  |  |
| **NHL** |  |  |  |  |  |  |  |  |  |  |  |  |  |  |  |  |  |  |  |  |
| Approach A q2^a^ |  | 1.022 |  | 0.944 | 1.106 |  | 1.013 |  | 0.939 | 1.092 |  | 1.049 |  | 0.972 | 1.132 |  | 1.050 |  | 0.974 | 1.131 |
| q3 |  | 1.032 |  | 0.952 | 1.119 |  | 1.061 |  | 0.986 | 1.142 |  | 1.029 |  | 0.953 | 1.112 |  | 1.058 |  | 0.982 | 1.139 |
| q4 |  | 0.994 |  | 0.915 | 1.080 |  | 0.987 |  | 0.919 | 1.059 |  | 1.076 |  | 0.997 | 1.161 |  | **1.092** |  | **1.018** | **1.170** |
| Trend test ^b^ |  | 0.997 |  | 0.971 | 1.024 |  | 0.997 |  | 0.975 | 1.020 |  | 1.021 |  | 0.996 | 1.046 |  | **1.027** |  | **1.005** | **1.050** |
| Approach B SPDE^c^ |  | 0.994 |  | 0.915 | 1.080 |  | 0.984 |  | 0.909 | 1.067 |  | 1.062 |  | 0.985 | 1.132 |  | 1.064 |  | 0.988 | 1.143 |
|  |  |  |  |  |  |  |  |  |  |  |  |  |  |  |  |  |  |  |  |  |
| **Myeloma** |  |  |  |  |  |  |  |  |  |  |  |  |  |  |  |  |  |  |  |  |
| Approach A q2^a^ |  | 1.045 |  | 0.967 | 1.131 |  | 1.043 |  | 0.967 | 1.126 |  | 1.002 |  | 0.92 | 1.092 |  | 1.012 |  | 0.936 | 1.094 |
| q3 |  | 0.990 |  | 0.916 | 1.071 |  | 1.006 |  | 0.932 | 1.086 |  | 0.987 |  | 0.903 | 1.077 |  | 1.022 |  | 0.946 | 1.104 |
| q4 |  | 0.977 |  | 0.910 | 1.050 |  | 0.970 |  | 0.904 | 1.040 |  | 0.979 |  | 0.898 | 1.065 |  | 0.979 |  | 0.910 | 1.052 |
| Trend test ^b^ |  | 0.987 |  | 0.966 | 1.009 |  | 0.985 |  | 0.964 | 1.007 |  | 0.992 |  | 0.965 | 1.019 |  | 0.992 |  | 0.969 | 1.015 |
| Approach B SPDE^c^ |  | 0.991 |  | 0.923 | 1.056 |  | 0.979 |  | 0.894 | 1.050 |  | 0.988 |  | 0.911 | 1.076 |  | 0.977 |  | 0.900 | 1.070 |
|  |  |  |  |  |  |  |  |  |  |  |  |  |  |  |  |  |  |  |  |  |
| **Leukemias** |  |  |  |  |  |  |  |  |  |  |  |  |  |  |  |  |  |  |  |  |
| Approach A q2^a^ |  | 0.969 |  | 0.912 | 1.03 |  | 0.958 |  | 0.904 | 1.016 |  | 1.065 |  | 0.997 | 1.138 |  | 1.023 |  | 0.961 | 1.09 |
| q3 |  | 0.984 |  | 0.925 | 1.047 |  | 1.005 |  | 0.95 | 1.064 |  | 0.966 |  | 0.902 | 1.034 |  | 0.966 |  | 0.905 | 1.029 |
| q4 |  | 1.025 |  | 0.964 | 1.089 |  | 1.033 |  | 0.980 | 1.090 |  | 1.015 |  | 0.950 | 1.084 |  | 0.990 |  | 0.930 | 1.051 |
| Trend test ^b^ |  | 1.011 |  | 0.992 | 1.031 |  | 1.016 |  | 0.999 | 1.034 |  | 0.996 |  | 0.976 | 1.017 |  | 0.992 |  | 0.973 | 1.012 |
| Approach B SPDE^c^ |  | 1.021 |  | 0.958 | 1.083 |  | 1.016 |  | 0.955 | 1.080 |  | 0.982 |  | 0.922 | 1.053 |  | 0.975 |  | 0.914 | 1.048 |
|  |  |  |  |  |  |  |  |  |  |  |  |  |  |  |  |  |  |  |  |  |

^a^ Quartiles: reference [ 6.46, 20.2); q2 [20.24, 25.0); q3 [24.99, 29.9); q4 [29.86,243.7] mg kg^-1^.

^b^ RR, taking quartiles as a categorical variable

^c^ RR for a change of one unit in the logarithm of the elements' soil concentration

Table S3. Summary of estimates of the effect (RR) of **arsenic** **topsoil levels**, categorised in quartiles, on mortality due to different tumour types, by sex. The table shows the results of the approaches A and B, broken down as follows: unadjusted (model n.1); and adjusted for socio-demographic variables and industrial emissions (model n.3).

|  |  | Men | | | | | | | | |  | Women | | | | | | | | |
| --- | --- | --- | --- | --- | --- | --- | --- | --- | --- | --- | --- | --- | --- | --- | --- | --- | --- | --- | --- | --- |
|  |  | Unadjusted | | | |  | Adjusted | | | |  | Unadjusted | | | |  | Adjusted | | | |
| Cancer site |  | RR |  | 95% | CI |  | RR |  | 95% | CI |  | RR |  | 95% | CI |  | RR |  | 95% | CI |
| **Buccal cavity**  **and pharynx** |  |  |  |  |  |  |  |  |  |  |  |  |  |  |  |  |  |  |  |  |
| Approach A q2^a^ |  | 1.003 |  | 0.937 | 1.073 |  | 0.987 |  | 0.922 | 1.057 |  | 1.102 |  | 0.986 | 1.233 |  | 1.069 |  | 0.958 | 1.194 |
| q3 |  | 1.018 |  | 0.943 | 1.099 |  | 1.048 |  | 0.977 | 1.123 |  | 1.042 |  | 0.918 | 1.183 |  | 1.050 |  | 0.931 | 1.184 |
| q4 |  | **1.100** |  | **1.018** | **1.189** |  | **1.232** |  | **1.158** | **1.310** |  | 1.076 |  | 0.957 | 1.211 |  | 1.051 |  | 0.947 | 1.165 |
| Trend test ^b^ |  | **1.033** |  | **1.006** | **1.059** |  | **1.076** |  | **1.054** | **1.097** |  | 1.018 |  | 0.980 | 1.057 |  | 1.012 |  | 0.979 | 1.046 |
| Approach B SPDE^c^ |  | **1.081** |  | **1.025** | **1.149** |  | **1.081** |  | **1.024** | **1.147** |  | 1.053 |  | 0.974 | 1.144 |  | 1.048 |  | 0.971 | 1.137 |
|  |  |  |  |  |  |  |  |  |  |  |  |  |  |  |  |  |  |  |  |  |
| **Esophagus** |  |  |  |  |  |  |  |  |  |  |  |  |  |  |  |  |  |  |  |  |
| Approach A q2^a^ |  | 0.976 |  | 0.91 | 1.046 |  | 0.951 |  | 0.887 | 1.021 |  | 0.92 |  | 0.789 | 1.073 |  | 0.865 |  | 0.738 | 1.012 |
| q3 |  | 0.959 |  | 0.887 | 1.038 |  | 1.004 |  | 0.935 | 1.078 |  | 0.912 |  | 0.766 | 1.085 |  | 0.925 |  | 0.783 | 1.091 |
| q4 |  | 1.030 |  | 0.952 | 1.115 |  | **1.148** |  | **1.077** | **1.224** |  | 1.020 |  | 0.866 | 1.198 |  | 1.092 |  | 0.948 | 1.26 |
| Trend test ^b^ |  | 1.010 |  | 0.984 | 1.037 |  | **1.052** |  | **1.030** | **1.074** |  | 1.010 |  | 0.957 | 1.066 |  | 1.043 |  | 0.996 | 1.093 |
| Approach B SPDE^c^ |  | 1.032 |  | 0.978 | 1.095 |  | 1.026 |  | 0.971 | 1.088 |  | 1.052 |  | 0.938 | 1.178 |  | 1.042 |  | 0.926 | 1.167 |
|  |  |  |  |  |  |  |  |  |  |  |  |  |  |  |  |  |  |  |  |  |
| **Stomach** |  |  |  |  |  |  |  |  |  |  |  |  |  |  |  |  |  |  |  |  |
| Approach A q2^a^ |  | 1.012 |  | 0.965 | 1.062 |  | 1.005 |  | 0.957 | 1.056 |  | 0.980 |  | 0.925 | 1.039 |  | 0.994 |  | 0.939 | 1.053 |
| q3 |  | 1.002 |  | 0.947 | 1.060 |  | 1.012 |  | 0.962 | 1.064 |  | 0.974 |  | 0.911 | 1.041 |  | 0.996 |  | 0.939 | 1.057 |
| q4 |  | 1.054 |  | 0.994 | 1.117 |  | **1.087** |  | **1.039** | **1.138** |  | 0.990 |  | 0.925 | 1.06 |  | **1.072** |  | **1.017** | **1.131** |
| Trend test ^b^ |  | 1.017 |  | 0.997 | 1.036 |  | **1.028** |  | **1.012** | **1.043** |  | 0.997 |  | 0.975 | 1.02 |  | **1.022** |  | **1.004** | **1.040** |
| Approach B SPDE^c^ |  | 1.035 |  | 0.994 | 1.079 |  | 1.028 |  | 0.986 | 1.073 |  | 1.015 |  | 0.970 | 1.069 |  | 1.014 |  | 0.964 | 1.064 |
|  |  |  |  |  |  |  |  |  |  |  |  |  |  |  |  |  |  |  |  |  |
| **Colorectal** |  |  |  |  |  |  |  |  |  |  |  |  |  |  |  |  |  |  |  |  |
| Approach A q2^a^ |  | **1.062** |  | **1.021** | **1.104** |  | 1.029 |  | 0.995 | 1.065 |  | 1.010 |  | 0.972 | 1.05 |  | 1.011 |  | 0.976 | 1.047 |
| q3 |  | **1.051** |  | **1.006** | **1.098** |  | **1.050** |  | **1.014** | **1.087** |  | 0.990 |  | 0.948 | 1.034 |  | 1.001 |  | 0.966 | 1.038 |
| q4 |  | **1.077** |  | **1.029** | **1.127** |  | **1.093** |  | **1.059** | **1.128** |  | 1.004 |  | 0.96 | 1.049 |  | 1.004 |  | 0.972 | 1.037 |
| Trend test ^b^ |  | **1.022** |  | **1.007** | **1.037** |  | **1.030** |  | **1.019** | **1.040** |  | 1.000 |  | 0.985 | 1.014 |  | 1.000 |  | 0.99 | 1.011 |
| Approach B SPDE^c^ |  | **1.042** |  | **1.009** | **1.080** |  | 1.026 |  | 0.996 | 1.057 |  | 1.008 |  | 0.976 | 1.039 |  | 0.996 |  | 0.968 | 1.026 |
|  |  |  |  |  |  |  |  |  |  |  |  |  |  |  |  |  |  |  |  |  |
| **Liver** |  |  |  |  |  |  |  |  |  |  |  |  |  |  |  |  |  |  |  |  |
| Approach A q2^a^ |  | 0.995 |  | 0.913 | 1.083 |  | 0.866 |  | 0.801 | 0.936 |  | 1.082 |  | 0.957 | 1.224 |  | 0.881 |  | 0.781 | 0.992 |
| q3 |  | 0.987 |  | 0.896 | 1.087 |  | 0.907 |  | 0.838 | 0.981 |  | 0.953 |  | 0.827 | 1.097 |  | 0.863 |  | 0.762 | 0.978 |
| q4 |  | **1.136** |  | **1.028** | **1.255** |  | 1.063 |  | 0.99 | 1.141 |  | 1.059 |  | 0.918 | 1.223 |  | 0.928 |  | 0.831 | 1.036 |
| Trend test ^b^ |  | **1.042** |  | **1.008** | **1.077** |  | **1.027** |  | **1.003** | **1.051** |  | 1.009 |  | 0.963 | 1.058 |  | 0.979 |  | 0.945 | 1.015 |
| Approach B SPDE^c^ |  | **1.087** |  | **1.013** | **1.180** |  | 1.048 |  | 0.979 | 1.135 |  | 0.984 |  | 0.893 | 1.087 |  | 0.938 |  | 0.856 | 1.042 |
|  |  |  |  |  |  |  |  |  |  |  |  |  |  |  |  |  |  |  |  |  |
| **Gallbladder** |  |  |  |  |  |  |  |  |  |  |  |  |  |  |  |  |  |  |  |  |
| Approach A q2^a^ |  | 1.025 |  | 0.926 | 1.134 |  | 0.967 |  | 0.871 | 1.072 |  | 0.928 |  | 0.855 | 1.007 |  | 0.931 |  | 0.862 | 1.006 |
| q3 |  | 1.102 |  | 0.985 | 1.235 |  | 1.063 |  | 0.953 | 1.183 |  | 0.954 |  | 0.868 | 1.048 |  | 0.941 |  | 0.867 | 1.022 |
| q4 |  | 1.021 |  | 0.914 | 1.142 |  | 0.923 |  | 0.835 | 1.019 |  | 0.901 |  | 0.819 | 0.992 |  | 0.812 |  | 0.754 | 0.876 |
| Trend test ^b^ |  | 1.010 |  | 0.974 | 1.047 |  | 0.981 |  | 0.95 | 1.012 |  | 0.970 |  | 0.940 | 1.001 |  | 0.938 |  | 0.915 | 0.960 |
| Approach B SPDE^c^ |  | 1.009 |  | 0.938 | 1.103 |  | 0.989 |  | 0.917 | 1.073 |  | 0.964 |  | 0.903 | 1.035 |  | 0.996 |  | 0.910 | 1.145 |
|  |  |  |  |  |  |  |  |  |  |  |  |  |  |  |  |  |  |  |  |  |
| **Pancreas** |  |  |  |  |  |  |  |  |  |  |  |  |  |  |  |  |  |  |  |  |
| Approach A q2^a^ |  | 1.044 |  | 0.991 | 1.099 |  | 1.029 |  | 0.979 | 1.081 |  | 1.050 |  | 0.992 | 1.111 |  | 1.023 |  | 0.971 | 1.078 |
| q3 |  | 0.989 |  | 0.932 | 1.049 |  | 1.001 |  | 0.95 | 1.055 |  | 1.029 |  | 0.966 | 1.097 |  | 1.040 |  | 0.984 | 1.099 |
| q4 |  | 1.06 |  | 0.999 | 1.124 |  | **1.092** |  | **1.043** | **1.144** |  | 1.053 |  | 0.988 | 1.122 |  | **1.059** |  | **1.009** | **1.112** |
| Trend test ^b^ |  | 1.015 |  | 0.996 | 1.035 |  | **1.027** |  | **1.012** | **1.043** |  | 1.014 |  | 0.993 | 1.035 |  | **1.019** |  | **1.003** | **1.035** |
| Approach B SPDE^c^ |  | **1.042** |  | **1.000** | **1.095** |  | 1.029 |  | 0.991 | 1.069 |  | 1.010 |  | 0.966 | 1.056 |  | 1.002 |  | 0.961 | 1.047 |
|  |  |  |  |  |  |  |  |  |  |  |  |  |  |  |  |  |  |  |  |  |
| **Peritoneum** |  |  |  |  |  |  |  |  |  |  |  |  |  |  |  |  |  |  |  |  |
| Approach A q2^a^ |  | 1.129 |  | 0.912 | 1.400 |  | 1.101 |  | 0.883 | 1.376 |  | 0.925 |  | 0.77 | 1.111 |  | 0.969 |  | 0.817 | 1.147 |
| q3 |  | 1.059 |  | 0.842 | 1.332 |  | 1.048 |  | 0.824 | 1.332 |  | 0.903 |  | 0.732 | 1.113 |  | 0.963 |  | 0.793 | 1.166 |
| q4 |  | 1.189 |  | 0.979 | 1.451 |  | 1.149 |  | 0.933 | 1.421 |  | 0.925 |  | 0.769 | 1.114 |  | 0.988 |  | 0.845 | 1.154 |
| Trend test ^b^ |  | 1.05 |  | 0.987 | 1.118 |  | 1.039 |  | 0.973 | 1.111 |  | 0.976 |  | 0.919 | 1.038 |  | 0.997 |  | 0.948 | 1.048 |
| Approach B SPDE^c^ |  | 1.134 |  | 0.995 | 1.305 |  | 1.110 |  | 0.968 | 1.288 |  | 0.965 |  | 0.852 | 1.094 |  | 0.986 |  | 0.866 | 1.116 |
|  |  |  |  |  |  |  |  |  |  |  |  |  |  |  |  |  |  |  |  |  |
| **Nasal cavity** |  |  |  |  |  |  |  |  |  |  |  |  |  |  |  |  |  |  |  |  |
| Approach A q2^a^ |  | 1.042 |  | 0.792 | 1.370 |  | 1.061 |  | 0.802 | 1.406 |  | 1.101 |  | 0.78 | 1.549 |  | 1.073 |  | 0.751 | 1.529 |
| q3 |  | 1.104 |  | 0.822 | 1.479 |  | 1.241 |  | 0.93 | 1.655 |  | 1.121 |  | 0.764 | 1.628 |  | 1.144 |  | 0.765 | 1.693 |
| q4 |  | 1.132 |  | 0.859 | 1.486 |  | **1.506** |  | **1.179** | **1.932** |  | 1.024 |  | 0.751 | 1.398 |  | 0.94 |  | 0.673 | 1.316 |
| Trend test ^b^ |  | 1.043 |  | 0.953 | 1.138 |  | **1.156** |  | **1.069** | **1.250** |  | 1.005 |  | 0.911 | 1.109 |  | 0.978 |  | 0.88 | 1.087 |
| Approach B SPDE^c^ |  | **1.219** |  | **1.027** | **1.412** |  | **2.303** |  | **1.814** | **2.709** |  | 0.905 |  | 0.769 | 1.127 |  | **1.913** |  | **1.459** | **2.316** |
|  |  |  |  |  |  |  |  |  |  |  |  |  |  |  |  |  |  |  |  |  |
| **Larynx** |  |  |  |  |  |  |  |  |  |  |  |  |  |  |  |  |  |  |  |  |
| Approach A q2^a^ |  | 0.951 |  | 0.89 | 1.016 |  | 0.928 |  | 0.868 | 0.991 |  | 0.975 |  | 0.773 | 1.227 |  | 0.973 |  | 0.766 | 1.233 |
| q3 |  | 0.963 |  | 0.891 | 1.040 |  | 0.931 |  | 0.868 | 0.997 |  | 0.873 |  | 0.665 | 1.137 |  | 0.914 |  | 0.689 | 1.204 |
| q4 |  | 1.013 |  | 0.937 | 1.096 |  | 1.014 |  | 0.953 | 1.079 |  | 1.096 |  | 0.897 | 1.34 |  | 1.04 |  | 0.838 | 1.293 |
| Trend test ^b^ |  | 1.006 |  | 0.98 | 1.032 |  | 1.007 |  | 0.987 | 1.027 |  | 1.028 |  | 0.963 | 1.098 |  | 1.013 |  | 0.944 | 1.087 |
| Approach B SPDE^c^ |  | 1.011 |  | 0.955 | 1.068 |  | 1.012 |  | 0.958 | 1.071 |  | 1.053 |  | 0.888 | 1.216 |  | **1.541** |  | **1.259** | **1.783** |
|  |  |  |  |  |  |  |  |  |  |  |  |  |  |  |  |  |  |  |  |  |
| **Lung** |  |  |  |  |  |  |  |  |  |  |  |  |  |  |  |  |  |  |  |  |
| Approach A q2^a^ |  | 0.994 |  | 0.962 | 1.027 |  | 0.99 |  | 0.961 | 1.019 |  | **1.072** |  | **1.003** | **1.146** |  | 1.036 |  | 0.977 | 1.098 |
| q3 |  | 1.001 |  | 0.962 | 1.041 |  | 1.004 |  | 0.974 | 1.034 |  | **1.109** |  | **1.030** | **1.194** |  | **1.087** |  | **1.023** | **1.154** |
| q4 |  | 1.018 |  | 0.976 | 1.062 |  | **1.044** |  | **1.015** | **1.073** |  | **1.133** |  | **1.050** | **1.222** |  | **1.129** |  | **1.069** | **1.191** |
| Trend test ^b^ |  | 1.006 |  | 0.992 | 1.021 |  | **1.015** |  | **1.006** | **1.024** |  | **1.041** |  | **1.015** | **1.067** |  | **1.042** |  | **1.024** | **1.060** |
| Approach B SPDE^c^ |  | 1.014 |  | 0.982 | 1.049 |  | 1.008 |  | 0.978 | 1.039 |  | **1.058** |  | **1.001** | **1.114** |  | 1.037 |  | 0.976 | 1.089 |
|  |  |  |  |  |  |  |  |  |  |  |  |  |  |  |  |  |  |  |  |  |
| **Pleura** |  |  |  |  |  |  |  |  |  |  |  |  |  |  |  |  |  |  |  |  |
| Approach A q2^a^ |  | 1.068 |  | 0.865 | 1.319 |  | 0.9 |  | 0.729 | 1.11 |  | 1.161 |  | 0.888 | 1.518 |  | 1.039 |  | 0.821 | 1.312 |
| q3 |  | 0.945 |  | 0.741 | 1.203 |  | 0.923 |  | 0.741 | 1.146 |  | 1.023 |  | 0.75 | 1.386 |  | 1.158 |  | 0.89 | 1.5 |
| q4 |  | 1.166 |  | 0.916 | 1.483 |  | 1.041 |  | 0.858 | 1.263 |  | 1.068 |  | 0.802 | 1.419 |  | 1.057 |  | 0.852 | 1.314 |
| Trend test ^b^ |  | 1.044 |  | 0.964 | 1.130 |  | 1.021 |  | 0.959 | 1.087 |  | 1.01 |  | 0.921 | 1.106 |  | 1.021 |  | 0.953 | 1.093 |
| Approach B SPDE^c^ |  | 1.123 |  | 0.954 | 1.342 |  | 1.048 |  | 0.893 | 1.245 |  | 0.676 |  | 0.584 | 0.837 |  | 1.005 |  | 0.836 | 1.21 |
|  |  |  |  |  |  |  |  |  |  |  |  |  |  |  |  |  |  |  |  |  |
| **Bone** |  |  |  |  |  |  |  |  |  |  |  |  |  |  |  |  |  |  |  |  |
| Approach A q2^a^ |  | 0.983 |  | 0.850 | 1.136 |  | 0.995 |  | 0.856 | 1.155 |  | 1.113 |  | 0.943 | 1.312 |  | 1.098 |  | 0.925 | 1.301 |
| q3 |  | 0.942 |  | 0.798 | 1.110 |  | 0.962 |  | 0.809 | 1.14 |  | 1.064 |  | 0.881 | 1.282 |  | 1.043 |  | 0.857 | 1.266 |
| q4 |  | 0.986 |  | 0.864 | 1.126 |  | 0.996 |  | 0.864 | 1.148 |  | 1.012 |  | 0.869 | 1.18 |  | 0.988 |  | 0.839 | 1.163 |
| Trend test ^b^ |  | 0.994 |  | 0.952 | 1.037 |  | 0.997 |  | 0.953 | 1.044 |  | 0.998 |  | 0.951 | 1.048 |  | 0.989 |  | 0.939 | 1.042 |
| Approach B SPDE^c^ |  | 1.011 |  | 0.924 | 1.112 |  | 1.028 |  | 0.931 | 1.136 |  | 0.977 |  | 0.877 | 1.083 |  | 0.956 |  | 0.851 | 1.067 |
|  |  |  |  |  |  |  |  |  |  |  |  |  |  |  |  |  |  |  |  |  |
| **Connective tissue** |  |  |  |  |  |  |  |  |  |  |  |  |  |  |  |  |  |  |  |  |
| Approach A q2^a^ |  | 0.966 |  | 0.847 | 1.103 |  | 0.956 |  | 0.841 | 1.085 |  | 0.934 |  | 0.803 | 1.085 |  | 0.881 |  | 0.75 | 1.034 |
| q3 |  | 0.902 |  | 0.776 | 1.046 |  | 0.911 |  | 0.787 | 1.052 |  | 0.989 |  | 0.836 | 1.168 |  | 0.990 |  | 0.838 | 1.169 |
| q4 |  | 1.024 |  | 0.899 | 1.163 |  | 1.043 |  | 0.928 | 1.173 |  | 1.001 |  | 0.859 | 1.168 |  | 1.066 |  | 0.922 | 1.232 |
| Trend test ^b^ |  | 1.006 |  | 0.963 | 1.049 |  | 1.015 |  | 0.977 | 1.054 |  | 1.006 |  | 0.957 | 1.058 |  | 1.035 |  | 0.988 | 1.084 |
| Approach B SPDE^c^ |  | 1.021 |  | 0.929 | 1.113 |  | 1.031 |  | 0.951 | 1.129 |  | 0.985 |  | 0.882 | 1.088 |  | 0.977 |  | 0.875 | 1.086 |
|  |  |  |  |  |  |  |  |  |  |  |  |  |  |  |  |  |  |  |  |  |
| **Melanoma** |  |  |  |  |  |  |  |  |  |  |  |  |  |  |  |  |  |  |  |  |
| Approach A q2^a^ |  | 1.008 |  | 0.907 | 1.121 |  | 0.970 |  | 0.883 | 1.065 |  | 1.067 |  | 0.957 | 1.190 |  | 1.064 |  | 0.960 | 1.18 |
| q3 |  | 0.991 |  | 0.879 | 1.118 |  | 0.959 |  | 0.863 | 1.064 |  | 1.016 |  | 0.898 | 1.149 |  | 1.039 |  | 0.926 | 1.166 |
| q4 |  | 1.101 |  | 0.985 | 1.231 |  | 0.997 |  | 0.913 | 1.088 |  | 0.994 |  | 0.889 | 1.111 |  | 1.018 |  | 0.924 | 1.123 |
| Trend test ^b^ |  | 1.032 |  | 0.995 | 1.07 |  | 1.000 |  | 0.972 | 1.029 |  | 0.993 |  | 0.958 | 1.029 |  | 1.002 |  | 0.971 | 1.033 |
| Approach B SPDE^c^ |  | 1.058 |  | 0.983 | 1.148 |  | 1.030 |  | 0.962 | 1.101 |  | 1.002 |  | 0.919 | 1.079 |  | 1.010 |  | 0.946 | 1.085 |
|  |  |  |  |  |  |  |  |  |  |  |  |  |  |  |  |  |  |  |  |  |
| **Skin** |  |  |  |  |  |  |  |  |  |  |  |  |  |  |  |  |  |  |  |  |
| Approach A q2^a^ |  | 1.016 |  | 0.885 | 1.166 |  | 1.058 |  | 0.921 | 1.215 |  | 0.87 |  | 0.745 | 1.017 |  | 0.936 |  | 0.806 | 1.086 |
| q3 |  | 1.064 |  | 0.909 | 1.246 |  | 1.023 |  | 0.882 | 1.188 |  | 0.983 |  | 0.824 | 1.171 |  | 0.954 |  | 0.815 | 1.117 |
| q4 |  | 1.048 |  | 0.901 | 1.219 |  | 1.009 |  | 0.884 | 1.152 |  | 0.962 |  | 0.81 | 1.141 |  | 0.993 |  | 0.865 | 1.14 |
| Trend test ^b^ |  | 1.017 |  | 0.969 | 1.068 |  | 0.999 |  | 0.957 | 1.042 |  | 0.997 |  | 0.941 | 1.055 |  | 1.002 |  | 0.958 | 1.047 |
| Approach B SPDE^c^ |  | 1.036 |  | 0.937 | 1.155 |  | 1.059 |  | 0.959 | 1.184 |  | 1.066 |  | 0.945 | 1.202 |  | 1.095 |  | 0.977 | 1.227 |
|  |  |  |  |  |  |  |  |  |  |  |  |  |  |  |  |  |  |  |  |  |
| **Breast** |  |  |  |  |  |  |  |  |  |  |  |  |  |  |  |  |  |  |  |  |
| Approach A q2^a^ |  |  |  |  |  |  |  |  |  |  |  | 1.011 |  | 0.972 | 1.051 |  | 0.989 |  | 0.954 | 1.025 |
| q3 |  |  |  |  |  |  |  |  |  |  |  | 1.042 |  | 0.997 | 1.088 |  | 1.026 |  | 0.989 | 1.065 |
| q4 |  |  |  |  |  |  |  |  |  |  |  | 1.012 |  | 0.967 | 1.058 |  | 0.964 |  | 0.931 | 0.997 |
| Trend test ^b^ |  |  |  |  |  |  |  |  |  |  |  | 1.005 |  | 0.990 | 1.02 |  | 0.990 |  | 0.980 | 1.001 |
| Approach B SPDE^c^ |  |  |  |  |  |  |  |  |  |  |  | 1.006 |  | 0.973 | 1.037 |  | 0.989 |  | 0.961 | 1.022 |
|  |  |  |  |  |  |  |  |  |  |  |  |  |  |  |  |  |  |  |  |  |
| **Uterus** |  |  |  |  |  |  |  |  |  |  |  |  |  |  |  |  |  |  |  |  |
| Approach A q2^a^ |  |  |  |  |  |  |  |  |  |  |  | 1.027 |  | 0.972 | 1.086 |  | 1.038 |  | 0.981 | 1.098 |
| q3 |  |  |  |  |  |  |  |  |  |  |  | 1.017 |  | 0.954 | 1.083 |  | 1.027 |  | 0.967 | 1.09 |
| q4 |  |  |  |  |  |  |  |  |  |  |  | 1.016 |  | 0.955 | 1.081 |  | 1.028 |  | 0.974 | 1.084 |
| Trend test ^b^ |  |  |  |  |  |  |  |  |  |  |  | 1.004 |  | 0.984 | 1.024 |  | 1.007 |  | 0.99 | 1.024 |
| Approach B SPDE^c^ |  |  |  |  |  |  |  |  |  |  |  | 1.033 |  | 0.990 | 1.077 |  | 1.023 |  | 0.980 | 1.067 |
|  |  |  |  |  |  |  |  |  |  |  |  |  |  |  |  |  |  |  |  |  |
| **Ovarian** |  |  |  |  |  |  |  |  |  |  |  |  |  |  |  |  |  |  |  |  |
| Approach A q2^a^ |  |  |  |  |  |  |  |  |  |  |  | 0.997 |  | 0.944 | 1.052 |  | 0.991 |  | 0.939 | 1.045 |
| q3 |  |  |  |  |  |  |  |  |  |  |  | 1.03 |  | 0.97 | 1.093 |  | 1.027 |  | 0.97 | 1.086 |
| q4 |  |  |  |  |  |  |  |  |  |  |  | 1.006 |  | 0.95 | 1.064 |  | 0.996 |  | 0.947 | 1.047 |
| Trend test ^b^ |  |  |  |  |  |  |  |  |  |  |  | 1.004 |  | 0.985 | 1.022 |  | 1.001 |  | 0.985 | 1.017 |
| Approach B SPDE^c^ |  |  |  |  |  |  |  |  |  |  |  | 1.004 |  | 0.967 | 1.050 |  | 0.992 |  | 0.953 | 1.040 |
|  |  |  |  |  |  |  |  |  |  |  |  |  |  |  |  |  |  |  |  |  |
| **Prostate** |  |  |  |  |  |  |  |  |  |  |  |  |  |  |  |  |  |  |  |  |
| Approach A q2^a^ |  | 1.005 |  | 0.967 | 1.044 |  | **1.036** |  | **1.000** | **1.074** |  |  |  |  |  |  |  |  |  |  |
| q3 |  | 0.99 |  | 0.948 | 1.033 |  | 1.027 |  | 0.989 | 1.065 |  |  |  |  |  |  |  |  |  |  |
| q4 |  | 0.984 |  | 0.942 | 1.029 |  | **1.054** |  | **1.019** | **1.091** |  |  |  |  |  |  |  |  |  |  |
| Trend test ^b^ |  | 0.994 |  | 0.98 | 1.008 |  | **1.016** |  | **1.005** | **1.027** |  |  |  |  |  |  |  |  |  |  |
| Approach B SPDE^c^ |  | 0.994 |  | 0.964 | 1.025 |  | 0.993 |  | 0.961 | 1.023 |  |  |  |  |  |  |  |  |  |  |
|  |  |  |  |  |  |  |  |  |  |  |  |  |  |  |  |  |  |  |  |  |
| **Bladder** |  |  |  |  |  |  |  |  |  |  |  |  |  |  |  |  |  |  |  |  |
| Approach A q2^a^ |  | 1.022 |  | 0.972 | 1.075 |  | 0.973 |  | 0.93 | 1.019 |  | 1.042 |  | 0.958 | 1.134 |  | 1.005 |  | 0.934 | 1.081 |
| q3 |  | 1.027 |  | 0.97 | 1.088 |  | 0.98 |  | 0.934 | 1.027 |  | 1.028 |  | 0.935 | 1.131 |  | 0.998 |  | 0.921 | 1.082 |
| q4 |  | 1.03 |  | 0.97 | 1.093 |  | 0.971 |  | 0.93 | 1.014 |  | 1.061 |  | 0.968 | 1.162 |  | 0.998 |  | 0.932 | 1.068 |
| Trend test ^b^ |  | 1.009 |  | 0.99 | 1.029 |  | 0.992 |  | 0.978 | 1.006 |  | 1.017 |  | 0.988 | 1.048 |  | 0.999 |  | 0.977 | 1.02 |
| Approach B SPDE^c^ |  | 1.022 |  | 0.98 | 1.073 |  | 1.005 |  | 0.965 | 1.045 |  | 1.048 |  | 0.987 | 1.121 |  | 1.02 |  | 0.965 | 1.086 |
|  |  |  |  |  |  |  |  |  |  |  |  |  |  |  |  |  |  |  |  |  |
| **Kidney** |  |  |  |  |  |  |  |  |  |  |  |  |  |  |  |  |  |  |  |  |
| Approach A q2^a^ |  | 1.051 |  | 0.98 | 1.127 |  | 0.983 |  | 0.919 | 1.051 |  | 0.969 |  | 0.886 | 1.061 |  | 0.933 |  | 0.861 | 1.011 |
| q3 |  | 1.077 |  | 0.996 | 1.166 |  | 1.044 |  | 0.972 | 1.119 |  | 0.978 |  | 0.883 | 1.082 |  | 0.982 |  | 0.899 | 1.073 |
| q4 |  | **1.132** |  | **1.047** | **1.223** |  | **1.094** |  | **1.027** | **1.164** |  | 0.985 |  | 0.894 | 1.084 |  | 0.97 |  | 0.9 | 1.045 |
| Trend test ^b^ |  | **1.041** |  | **1.015** | **1.068** |  | **1.035** |  | **1.014** | **1.056** |  | 0.997 |  | 0.966 | 1.028 |  | 0.996 |  | 0.973 | 1.02 |
| Approach B SPDE^c^ |  | **1.081** |  | **1.027** | **1.144** |  | 1.052 |  | 0.999 | 1.107 |  | 0.995 |  | 0.93 | 1.058 |  | 0.982 |  | 0.921 | 1.046 |
|  |  |  |  |  |  |  |  |  |  |  |  |  |  |  |  |  |  |  |  |  |
| **Brain** |  |  |  |  |  |  |  |  |  |  |  |  |  |  |  |  |  |  |  |  |
| Approach A q2^a^ |  | **1.071** |  | **1.007** | **1.139** |  | **1.069** |  | **1.006** | **1.137** |  | 1.063 |  | 0.989 | 1.142 |  | 1.037 |  | 0.966 | 1.114 |
| q3 |  | 1.03 |  | 0.962 | 1.103 |  | 1.050 |  | 0.983 | 1.121 |  | 1.057 |  | 0.975 | 1.146 |  | 1.076 |  | 0.998 | 1.161 |
| q4 |  | **1.118** |  | **1.049** | **1.190** |  | **1.135** |  | **1.071** | **1.202** |  | **1.093** |  | **1.012** | **1.18** |  | **1.107** |  | **1.036** | **1.184** |
| Trend test ^b^ |  | **1.033** |  | **1.012** | **1.054** |  | **1.038** |  | **1.02** | **1.057** |  | **1.027** |  | **1.002** | **1.053** |  | **1.035** |  | **1.013** | **1.057** |
| Approach B SPDE^c^ |  | **1.052** |  | **1.008** | **1.099** |  | **1.050** |  | **1.003** | **1.097** |  | **1.057** |  | **1.004** | **1.114** |  | 1.051 |  | 0.997 | 1.107 |
|  |  |  |  |  |  |  |  |  |  |  |  |  |  |  |  |  |  |  |  |  |
| **Thyroid** |  |  |  |  |  |  |  |  |  |  |  |  |  |  |  |  |  |  |  |  |
| Approach A q2^a^ |  | 1.078 |  | 0.883 | 1.318 |  | 1.049 |  | 0.862 | 1.275 |  | 0.952 |  | 0.813 | 1.113 |  | 0.927 |  | 0.797 | 1.076 |
| q3 |  | 1.112 |  | 0.89 | 1.384 |  | 1.118 |  | 0.901 | 1.383 |  | 0.954 |  | 0.798 | 1.137 |  | 0.971 |  | 0.823 | 1.142 |
| q4 |  | 0.987 |  | 0.801 | 1.206 |  | 1.015 |  | 0.844 | 1.221 |  | 0.976 |  | 0.826 | 1.147 |  | 1.041 |  | 0.909 | 1.191 |
| Trend test ^b^ |  | 0.999 |  | 0.937 | 1.062 |  | 1.005 |  | 0.948 | 1.066 |  | 0.995 |  | 0.942 | 1.048 |  | 1.021 |  | 0.978 | 1.066 |
| Approach B SPDE^c^ |  | 1.074 |  | 0.956 | 1.23 |  | 1.064 |  | 0.939 | 1.224 |  | 1.015 |  | 0.908 | 1.135 |  | 1.01 |  | 0.897 | 1.128 |
|  |  |  |  |  |  |  |  |  |  |  |  |  |  |  |  |  |  |  |  |  |
| **NHL** |  |  |  |  |  |  |  |  |  |  |  |  |  |  |  |  |  |  |  |  |
| Approach A q2^a^ |  | 1.019 |  | 0.945 | 1.098 |  | 0.989 |  | 0.92 | 1.063 |  | **1.086** |  | **1.012** | **1.165** |  | 1.033 |  | 0.962 | 1.108 |
| q3 |  | 1.003 |  | 0.925 | 1.088 |  | 1.017 |  | 0.943 | 1.095 |  | 1.069 |  | 0.988 | 1.156 |  | **1.080** |  | **1.003** | **1.163** |
| q4 |  | 1.043 |  | 0.962 | 1.129 |  | **1.099** |  | **1.028** | **1.174** |  | **1.091** |  | **1.012** | **1.175** |  | **1.126** |  | **1.054** | **1.203** |
| Trend test ^b^ |  | 1.012 |  | 0.986 | 1.039 |  | **1.034** |  | **1.012** | **1.056** |  | 1.025 |  | 1.000 | 1.049 |  | **1.041** |  | **1.020** | **1.063** |
| Approach B SPDE^c^ |  | 1.032 |  | 0.976 | 1.087 |  | 1.019 |  | 0.961 | 1.074 |  | 1.049 |  | 0.998 | 1.108 |  | 1.035 |  | 0.986 | 1.09 |
|  |  |  |  |  |  |  |  |  |  |  |  |  |  |  |  |  |  |  |  |  |
| **Myeloma** |  |  |  |  |  |  |  |  |  |  |  |  |  |  |  |  |  |  |  |  |
| Approach A q2^a^ |  | 1.041 |  | 0.971 | 1.116 |  | 1.048 |  | 0.977 | 1.123 |  | 0.969 |  | 0.888 | 1.058 |  | 0.995 |  | 0.917 | 1.077 |
| q3 |  | 0.941 |  | 0.87 | 1.018 |  | 0.971 |  | 0.897 | 1.05 |  | 0.965 |  | 0.889 | 1.046 |  | 0.985 |  | 0.919 | 1.055 |
| q4 |  | 0.998 |  | 0.933 | 1.068 |  | 1.012 |  | 0.947 | 1.082 |  | 0.984 |  | 0.933 | 1.043 |  | 0.986 |  | 0.932 | 1.042 |
| Trend test ^b^ |  | 0.993 |  | 0.972 | 1.015 |  | 0.998 |  | 0.977 | 1.02 |  | 0.981 |  | 0.955 | 1.007 |  | 0.984 |  | 0.962 | 1.007 |
| Approach B SPDE^c^ |  | 1.001 |  | 0.954 | 1.045 |  | 1.007 |  | 0.962 | 1.055 |  | 0.981 |  | 0.955 | 1.007 |  | 0.984 |  | 0.962 | 1.007 |
|  |  |  |  |  |  |  |  |  |  |  |  |  |  |  |  |  |  |  |  |  |
| **Leukemias** |  |  |  |  |  |  |  |  |  |  |  |  |  |  |  |  |  |  |  |  |
| Approach A q2^a^ |  | 1.030 |  | 0.973 | 1.091 |  | 0.995 |  | 0.941 | 1.051 |  | 1.024 |  | 0.963 | 1.09 |  | 0.985 |  | 0.929 | 1.045 |
| q3 |  | 1.046 |  | 0.983 | 1.114 |  | 1.035 |  | 0.976 | 1.097 |  | 1.012 |  | 0.944 | 1.085 |  | 0.990 |  | 0.929 | 1.056 |
| q4 |  | **1.061** |  | **1.000** | **1.126** |  | 1.031 |  | 0.978 | 1.086 |  | 1.027 |  | 0.964 | 1.095 |  | 0.991 |  | 0.935 | 1.048 |
| Trend test ^b^ |  | **1.019** |  | **1.000** | **1.039** |  | 1.012 |  | 0.996 | 1.029 |  | 1.007 |  | 0.987 | 1.028 |  | 0.998 |  | 0.980 | 1.016 |
| Approach B SPDE^c^ |  | **1.045** |  | **1.005** | **1.088** |  | **1.040** |  | **1.000** | **1.087** |  | 1.011 |  | 0.970 | 1.061 |  | 1.003 |  | 0.960 | 1.054 |
|  |  |  |  |  |  |  |  |  |  |  |  |  |  |  |  |  |  |  |  |  |

^a^ Quartiles: reference [ 1.00. 9.11); q2 [ 9.11.12.82); q3 [12.82,16.97); q4 [16.97,99.37] mg kg^-1^.

^b^ RR, taking quartiles as a categorical variable

^c^ RR for a change of one unit in the logarithm of the elements' soil concentration
